# Soil, rhizosphere, and root microbiome in kiwifruit vine decline, an emerging multifactorial disease

**DOI:** 10.3389/fmicb.2024.1330865

**Published:** 2024-03-21

**Authors:** Micol Guaschino, Marco Garello, Luca Nari, Yeka V. Zhimo, Samir Droby, Davide Spadaro

**Affiliations:** ^1^Department of Agricultural, Forestry and Food Sciences (DiSAFA), University of Torino, Grugliasco, Italy; ^2^Interdepartmental Centre for Innovation in Agro-environmental Sector – AGROINNOVA, University of Turin, Grugliasco, Italy; ^3^Fondazione Agrion, Manta, Italy; ^4^Department of Postharvest Science, ARO, The Volcani Center, Rishon LeZion, Israel

**Keywords:** multifactorial disease, kiwifruit vine decline syndrome, microbiome, dysbiosis, *Phytopythium*, next-generation sequencing, metabarcoding

## Abstract

Kiwifruit vine decline syndrome (KVDS) is characterized by severe root system impairment, which leads to irreversible wilting of the canopy. Plants usually collapse rapidly from the appearance of the first aboveground symptoms, without recovery even in the following seasons. The syndrome has been negatively impacting kiwifruit yield in different areas of Italy, the main producing European country, since its first outbreak in 2012. To date, a unique, common causal factor has yet to be found, and the syndrome is referred to as multifactorial. In this article, we investigated the whole biotic community (fungi, bacteria, and oomycetes) associated with the development of KVDS in three different belowground matrices/compartments (soil, rhizosphere, and root). Sampling was performed at both healthy and affected sites located in the main kiwifruit-producing area of Northwestern Italy. To address the multifactorial nature of the syndrome and to investigate the potential roles of abiotic factors in shaping these communities, a physicochemical analysis of soils was also performed. This study investigates the associations among taxonomic groups composing the microbiome and also between biotic and abiotic factors. Dysbiosis was considered as a driving event in shaping KVDS microbial communities. The results obtained from this study highlight the role of the oomycete genus *Phytopythium*, which resulted predominantly in the oomycete community composition of diseased matrices, though it was also present in healthy ones. Both bacterial and fungal communities resulted in a high richness of genera and were highly correlated to the sampling site and matrix, underlining the importance of multiple location sampling both geographically and spatially. The rhizosphere community associated with KVDS was driven by a dysbiotic process. In addition, analysis of the association network in the diseased rhizosphere revealed the presence of potential cross-kingdom competition for plant-derived carbon between saprobes, oomycetes, and bacteria.

## 1 Introduction

In the framework of changing climate where average temperatures are rising and both the frequency and intensity of extreme weather events are expected to increase, co-occurring stress factors are becoming the main drivers of the severe decline in plant growth and survival (Zandalinas et al., 2021a). Woody plant declines are characterized by a degeneration of plant tissues brought on by multifactorial stress, which is defined as the combination of biotic, climate-driven, and/or soil-associated stress factors simultaneously impacting plant health, with a decrease in overall productivity (Bettenfeld et al., 2020; Zandalinas et al., 2021b). In the last decade, several perennials, including different woody plant species, have been increasingly affected by decline. Affected agroecosystems include vineyards (Fontaine et al., 2016; Gramaje et al., 2018; Merot et al., 2023), olive groves (Luvisi et al., 2017), peach (Yang et al., 2012), apple (Mazzola and Manici, 2012; Nicola et al., 2018), citrus (Ezrari et al., 2021), and kiwifruit orchards (Savian et al., 2020).

Global kiwifruit production totals approximately 3.5 million tons, with Italy ranking as the world’s third-largest producer, following China and New Zealand (FAOSTAT, n.d.). In 2022, Italy produced 523,120 tons of kiwifruit, of which 250,000 tons were exported (Balestra and Costa, 2020). Since 2012, a condition known as kiwifruit vine decline syndrome (KVDS) has affected over 10% (almost 2,900 hectares) of Italian kiwifruit orchards, spanning regions in the north (Veneto, Piedmont, Friuli Venezia Giulia), central (Lazio, Emilia Romagna), and southern Italy (Calabria) (Sorrenti et al., 2019). However, it is estimated that over 25% of Italian kiwifruit orchards are affected (Savian et al., 2020). KVDS symptoms lead to severe root decay and irreversible wilting, and plants usually die within a few weeks from the appearance of the first symptoms in the canopy. Aboveground symptoms include leaf curl, necrosis, and twig wilting, which appear long after impairment of the root system. Roots are characterized by central stele break-off, cortex detachment, rotting areas, and the disappearance of finer feeding roots (Donati et al., 2020). The main pathogens associated with disease development, based on isolation techniques, are mainly soil-borne oomycetes belonging to the genera *Phytopythium* and *Phytophthora*, although they cannot be considered as the exclusive causal factors (Tacconi et al., 2015; Bardi, 2020). Both oomycete genera are characterized by a lifestyle that requires water for zoospore spread and shows optimal growth at high temperature (Brasier et al., 2022; Prencipe et al., 2023); therefore, waterlogging and climate change are considered as co-factors for KVDS development. Kiwifruit has been reported to be sensitive to both root anoxia caused by flooding events and high transpiration demand, together with high soil temperatures (Save and Serrano, 1986; Smith et al., 1989). Donati et al. (2020) isolated bacterial, fungal, and oomycete communities from roots with different KVDS incidence located in Northeastern Italy, followed by inoculation of fungal and oomycete isolates on potted kiwi vines. In the same paper, the inoculation of *Phytopythium* spp., *Phytophthora* spp., *Desarmillaria tabescens*, and oomycete reference strains was performed together with the waterlogging of pots. The results showed the reproduction of symptoms by all the isolates, regardless of the irrigation strategy, suggesting a crucial role of multiple oomycete genera in the disease. In another recent study by Savian et al. (2020), the relationship between waterlogging, soil-borne pathogens, and KVDS was investigated. In this study, historical rainfall data from two locations in Northeastern Italy were analyzed and greenhouse trials designed to evaluate the contribution of waterlogging to KVDS onset were conducted. Inoculation of kiwi plantlets with naturally infected bacteria in combination with waterlogging permitted symptom reproduction. Pathogen isolation revealed a high occurrence of the species *Phytopythium vexans*, *Phytopythium chamaehyphon*, and *Fusarium solani* (Savian et al., 2020). *P. vexans* was also previously described as being pathogenic toward kiwifruit roots in Turkey (Polat et al., 2017). In Italy, inoculation of *P. vexans* isolates combined with waterlogging was performed in greenhouse trials by Prencipe et al. (2020) to confirm pathogenicity. Similarly, *P. chamaehyphon* pathogenicity was confirmed by Savian et al. (2021) on kiwifruit. In addition, oomycetes were isolated from KVDS-symptomatic plants in the northwestern area of Italy in 2016, 2017, and 2019 (Prencipe et al., 2023). Oomycetes belonging to the species *P. vexans*, *P. chamaehyphon*, *Phytopythium helicoides*, and *Phytopythium litorale* were recovered at the highest rates. In the same study, their pathogenicity in kiwifruit was verified (and Koch postulates satisfied) by symptom reproduction with waterlogging treatment.

*Phytopythium* spp. have also been reported more broadly as root rot causal agents on kiwifruit in different countries, like China (Wang et al., 2015), Turkey (Türkkan et al., 2022), and Japan (Shimizu et al., 2005). *Phytophthora* has also been isolated and identified as a kiwi root rot pathogen with symptoms similar to KVDS in France (Baudry et al., 1991) and Turkey (Akilli et al., 2011; Kurbetli and Ozan, 2013).

In recent years, more in-depth studies using next-generation sequencing (NGS) techniques have been performed with the objective of defining the whole biotic community associated with the syndrome. In Central Italy, biotic components of KVDS were characterized by Manici et al. (2022) combining isolation techniques for fungal endophytes with qPCR quantification (of fungal and bacterial DNA) and NGS techniques for soil bacterial composition analysis. The study evidenced a reduction of bacterial communities in diseased samples as contributing to reduced plant growth. In Northeastern Italy, Savian et al. (2022) used a metabarcoding approach to describe oomycete and fungal communities in the root endosphere and rhizosphere of healthy versus diseased samples, evidencing the role of *Phytophthora sojae* and *Phytopythium* spp. in KVDS.

Microbial populations (namely fungi, bacteria, oomycetes, protists, archaea, and viruses) are deeply influenced by geographic location, environmental properties (e.g., soil composition, plant host species/genotypes), and agronomic practices (Fitzpatrick et al., 2020). Moreover, woody plants and their associated microbiota, are particularly susceptible to global change factors (Bettenfeld et al., 2020). Climate change can impact soil properties and generate environmental fluctuations, which can affect the bulk soil microbiome, the main source/pool of potential plant-colonizing microorganisms. Microorganisms, in turn, can exert different responses to adverse conditions depending on lifestyle, infection strategy, and sensitivity to abiotic factors (Zandalinas et al., 2021a). In comparison to bulk soil, the rhizosphere (the first plant-influenced habitat) is actively shaped by plant factors including root morphology, immune response, physiological changes (e.g., adaptation responses to climate change), and root exudate production (Trivedi et al., 2022). In the root endosphere, the enrichment of microbial groups is influenced by all these factors and by the filtration effect of the plant immune system (Singh et al., 2020).

As previously mentioned, microbial populations are subjected to change, especially in conditions of long-term abiotic stress imposed by climate change. In particular, the expansion of niches for novel pathogens is favored by rising temperatures (Bebber et al., 2013; Cavicchioli et al., 2019). In this framework, plants exert less control over the associated microbiome, increasing the possibility for dysbiosis, leading to higher disease susceptibility of the plant and the inability to balance host-associated microbiome composition when external stressors are present. At the same time, in the interaction between the plant and its microbiome, community assembly and interactions may vary, determining stress resilience and resistance (Trivedi et al., 2022). The Anna Karenina Principle (AKP) proposes that the microbiome associated with dysbiotic communities is subjected to an increase in stochastic assembly processes, leading to more variability compared to healthy ones (Arnault et al., 2023).

The objective of the present study was to characterize the entire community associated with KVDS, considering bacteria, fungi, and oomycetes. The biotic component was characterized in three different belowground compartments (soil, rhizosphere, and roots) of kiwifruit orchards located in Northwest Italy. In addition to characterizing the communities, secondary aims were to determine the associations between taxa and to evaluate the possible involvement of a dysbiosis process in community assembly. To address the contribution of the abiotic component in the syndrome, association networks between the physicochemical characteristics of soils and rhizosphere taxa were assessed.

## 2 Materials and methods

### 2.1 Orchard location, sampling, and DNA extraction

**Figure 1:**
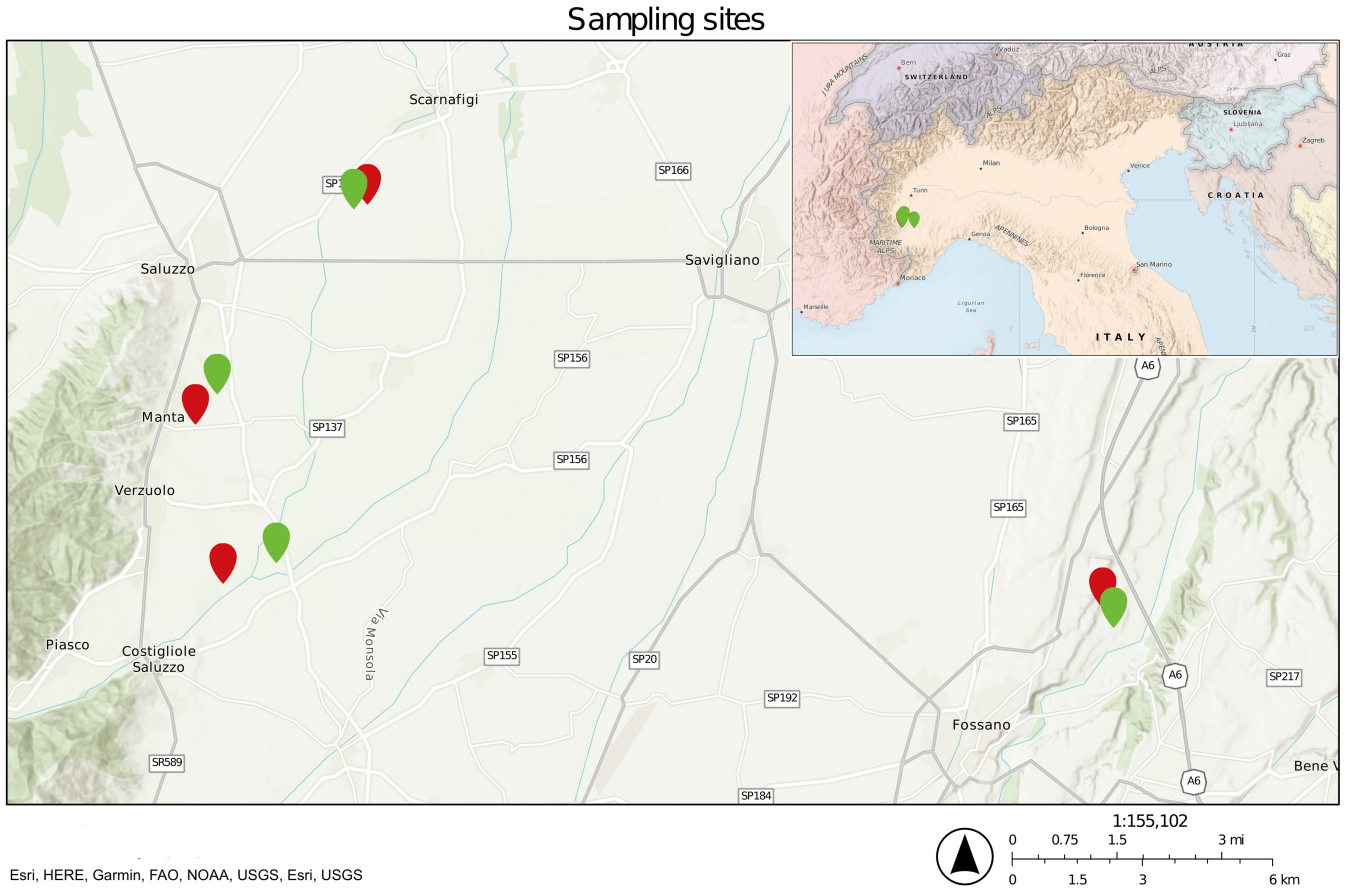
Sampling site map. Green pinpoints refer to healthy sites, whereas red pinpoints refer to diseased ones.

Eight sites in four separate locations in Cuneo province, northwest Italy, were selected to sample three different matrices (soil, rhizosphere, and root) both in diseased and healthy orchards (Figure 1 and Supplementary Table S1). From each sampling site, random plants were selected, and soil was collected at a depth of 30 cm after removing the topsoil. At the same time, roots and the attached soil were collected from different parts of the radical apparatus. The collected roots were symptomatic but without excessive necrosis. Each sample of soil, rhizosphere, and root was collected in five biological replicates and treated independently. Roots were further processed in the laboratory to detach the soil attached to the root surface, following a previously reported protocol (Simmons et al., 2018) with minor modifications.

Prior to DNA extraction, washed roots were crushed using mortar and pestle with liquid nitrogen. Further breakage of the roots was achieved with metal beads and TissueLyser II (Qiagen, Hilden, Germany). Total DNA extraction from all the belowground matrices was performed with the DNeasy PowerSoil kit (Qiagen, Hilden, Germany) following the manufacturer’s instructions. The extracted DNA was quantified and quality checked with the NanoDrop 2000 spectrophotometer (Thermo Fisher Scientific, Waltham, USA) and stored at −20°C.

### 2.2 Metabarcoding sequencing

Metabarcoding sequencing was performed by IGATech (Udine, Italy) with different primer sets for fungi, bacteria, and oomycetes. For bacteria, primers 341f (Klindworth et al., 2013) and 806r (Apprill et al., 2015) were selected. For both fungi and oomycetes, the reverse primer ITS4ngs (Tedersoo et al., 2014) was used, while the selected forward primers were fITS7 (Ihrmark et al., 2012) and ITS3oo (Riit et al., 2016, 2018), respectively. Libraries were prepared by following the Illumina 16S Metagenomic Sequencing Library Preparation Protocol (Illumina, 2013) in two amplification steps: (1) an initial PCR amplification using locus-specific PCR primers, and (2) a subsequent amplification that integrates relevant flow cell-binding domains and unique indices (Nextera XT Index Kit, FC-131-1001/FC-131-1002). Peptide nucleic acid (PNA) clamping was applied during the first amplification step to block the amplification of host chloroplast and mitochondrial 16S sequences following the manufacturer’s protocol (PNA Bio Inc., Newbury Park, CA). For the locus-specific amplification of 16S and ITS libraries, 35 and 29 cycles were applied in the first PCR reaction, respectively. Libraries were sequenced on NovaSeq6000 instruments (Illumina, San Diego, CA) using 250-bp paired-end mode. During the analysis, 15 samples associated with oomycetes did not result in a viable sequencing library and thus had to be dropped. Therefore, the total number of samples was 120 for fungi and bacteria and 105 for oomycetes.

### 2.3 Bioinformatics for taxonomic assignment and alpha and beta-diversity

Sequence analysis for oomycetes, fungi, and bacteria was performed separately using the QIIME2 suite (Bolyen et al., 2019), version 2021.2. Primer contamination removal was carried out using Cutadapt (Martin, 2011; via q2-cutadapt), while read merging, ASV calling and chimera removal were done using DADA2 (Callahan et al., 2016; via q2-dada2) with default settings. In this step, based on base quality value distribution, no. 5′ trimming was performed for either forward or reverse reads. Taxonomic assignment of ASVs was achieved using the q2-feature classifier (Bokulich et al., 2018) with a naive Bayes predictor. For fungi and oomycetes, the predictor was trained on the UNITE database, version 8.3-eukaryotes-global (Abarenkov et al., 2021), which was integrated with ITS sequences taken from the National Center for Biotechnology Information nucleotide database (NCBI).^1^ For bacteria, the predictor was trained on the SILVA database, version 138 (Glöckner et al., 2017), elaborated with the RESCRIPt plugin (Bokulich et al., 2023), after sequences were trimmed down to the V3V4 regions (via q2-features-classifier). In all three analyses, an ASV filtering step was performed: first matrix contaminations were removed (via q2-taxa), then ASVs with a frequency < 0.0001% of total ASVs were removed, as well as ASVs that appeared in less than five biological replications (for fungi and bacteria) or three biological replications (for oomycetes) (via q2-feature-table). Normalization for alpha and beta diversity was carried out with SRS (Heidrich et al., 2021) (via q2-srs), with a depth of 81,336 (fungi), 35,900 (bacteria), and 600 (oomycetes) ASVs. Shannon Index (Shannon and Weaver, 1949), number of observed features (DeSantis et al., 2006), and Pielou evenness (Pielou, 1966) were chosen as alpha diversity metrics and calculated with the default q2-diversity plugin, while robust Aitchison distance was selected as beta-diversity metric and calculated with DEICODE (Martino et al., 2019; via q2-deicode).

Statistical analyses of alpha diversity results were performed using the stats package R implementation of the non-parametric Kruskal–Wallis test (Kruskal and Wallis, 2012). Post-hoc analysis for statistically significant results was carried out with the Dunn post-hoc test as implemented in the FSA R package (Ogle et al., 2023), with Benjamini–Hochberg false discovery rate (FDR) correction (Benjamini and Hochberg, 1995). Beta-diversity results were used to carry out a Principal Coordinates Analysis (PCoA) with DEICODE (with the auto-RPCA method) and a Permutational Analysis of Variance (PERMANOVA) test using the Adonis plugin (Anderson, 2001) with 999 permutations. Differences in variance were assessed with the permadisp/betadisp test (Anderson, 2006), also with 999 permutations. Results of the PCoA were used to generate 2D plots by using (R Core Team, 2023) packages ggplot2 (Wickham, 2016), ggpubr (Kassambara, 2023), rcompanion (Mangiafico, 2023), and dplyr (Wickham et al., 2023).

### 2.4 Microbial community composition, association networks, and evaluation of ecological processes

Visualization of microbial community composition in the matrices was done by collapsing ASV absolute frequencies at the genus level and by converting them to relative frequencies. Differential abundance of taxa between different health statuses for each matrix was measured with ALDEx2 (Fernandes et al., 2013, 2014; Gloor et al., 2016), via the q2-aldex2 plugin. For visualization, statistically significant comparisons were selected where relative abundance was at least 1% in either thesis or change was at least 2-fold.

Association networks were calculated using the CoNet plugin (Faust and Raes, 2016) in combination with Cytoscape version 3.9.1 (Shannon et al., 2003) and visualized with Gephi (Bastian et al., 2009). A correlation cutoff of ±0.60 was selected to filter collinear variables.

The impact of stochastic and deterministic processes on microbiota composition was investigated by calculating the βNTI (beta Nearest Taxon Index) and the modified Raup-Crick metric based on Bray–Curtis dissimilarity, as described in a previous study (Stegen et al., 2013). For the Raup-Crick metric, an optimized script was employed (Richter-Heitmann et al., 2020). To calculate these metrics, representative sequences from each taxon were aligned with MAFFT (Katoh and Standley, 2013), and a phylogenetic tree was calculated with FastTree (Price et al., 2010). These results were plotted with packages ggplot2 and ggpubr.

### 2.5 Physicochemical analysis of soils

Soil samples for physicochemical analysis were collected from the same sites used for metabarcoding analysis. This was done by collecting 0.5 kg of soil from between 20 and 40 cm deep. For each site, three diagonal sampling points were pooled and sent to Laboratorio Agrochimico Regionale (LAR)^2^ of Piedmont Region (Torino, Italy) for analysis. The considered parameters were percentages of clay, loam, limestone, soil organic substance, and sand for soil composition. Soil water pH, organic carbon, and total nitrogen content in the soil were measured. Mineral exchanges and assimilations (ppm) were also calculated. For explorative visualization of the physicochemical data with dimensionality reduction, a non-metric multidimensional scaling (NMDS) was performed with the meta-MDS function of the vegan package (Oksanen et al., 2020), with Bray–Curtis as metric.

The physicochemical variables of the considered soils were included as meta-variables for interactions between biotic and abiotic factors in the association networks previously described.

### 2.6 qPCR quantification of *Phytopythium vexans* and oomycete isolation

Quantification of the target species, *P. vexans*, to confirm sequencing results was performed in three biological replicates for each sample considered. The quantification was performed by using *P. vexans* phylotype-specific primers and a qPCR protocol from Bent et al. (2009). Standard deviation for the sample quantification was calculated based on the three technical qPCR replicates. Culture-based isolation of oomycetes from the roots of diseased orchards was performed by using a semiselective substrate. Macro and micromorphological analyses were performed on the isolates. In addition, molecular identification was performed, as described by Prencipe et al. (2023).

## 3 Results

### 3.1 Metabarcoding sequencing

Overall, sequencing of 120 samples for fungi and bacteria and 105 samples for oomycetes resulted in 58,603,041 (fungi), 41,229,343 (bacteria), and 5,205,191 (oomycetes) sequences associated with 20,306, 150,349, and 3,414 features, respectively. Subsequent filtering of contaminants and very low abundance of ASVs resulted in 50,916,370 (fungi), 20,992,728 (bacteria), and 2,223,433 (oomycetes) sequences associated with 893, 1,711, and 190 features, respectively. On average, each sample represented 424,303, 174,939, and 21,175 sequences, respectively. Sequences were deposited in the European Nucleotide Archive (ENA)^3^ database under project accession PRJEB70619.

### 3.2 Alpha and beta diversity

Alpha- (Supplementary Table S2) and beta-diversity indexes were calculated for each matrix for bacteria, fungi, and oomycetes.

**Figure 2:**
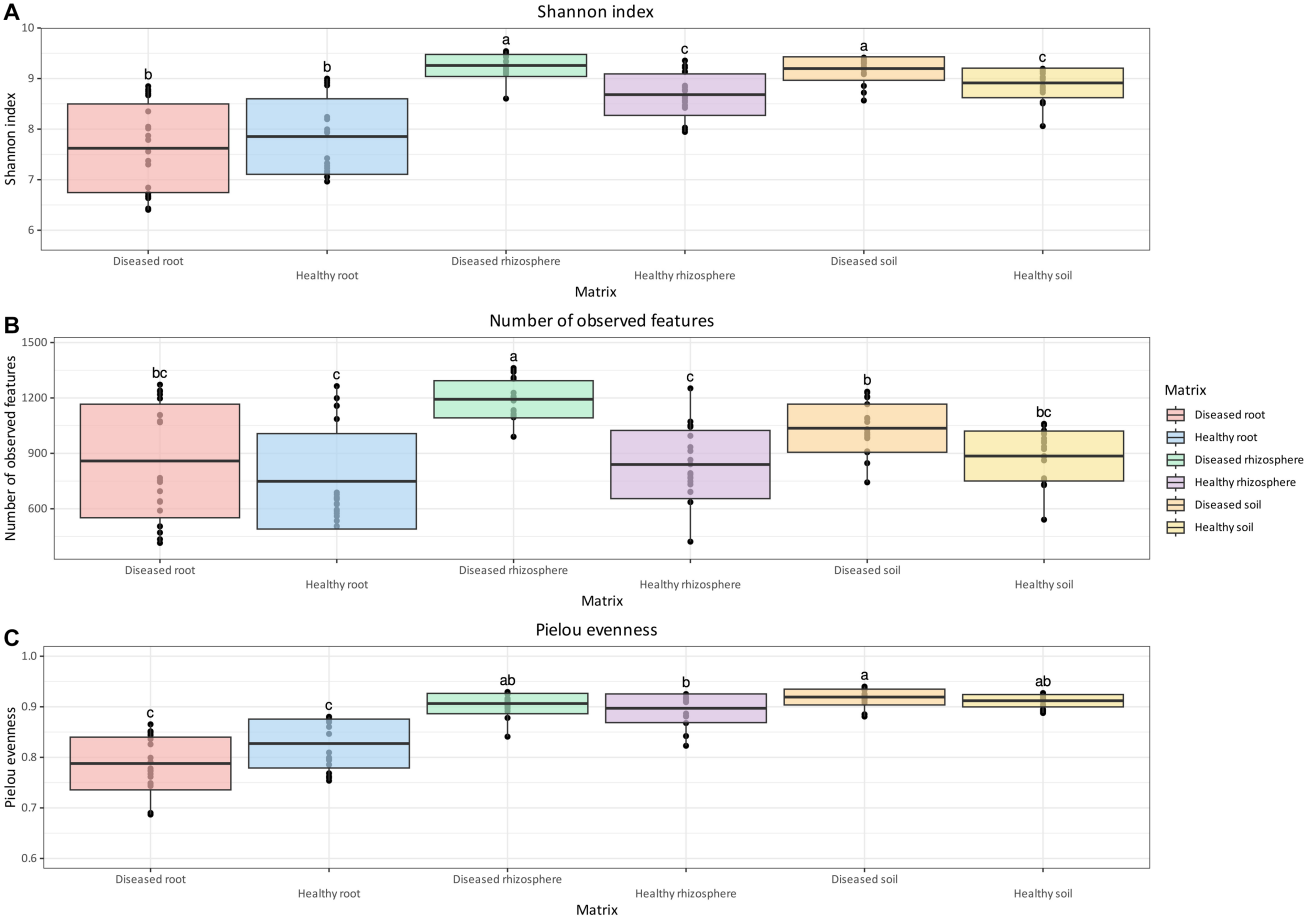
Box and whisker plot of Shannon Index **(A)**, number of observed features **(B)**, and Pielou evenness **(C)** measured across matrix and health status for bacteria. Whiskers extend to ±1.5 interquartile range. The presence of statistically significant differences was assessed by means of a Kruskal–Wallis test, followed by a Dunn *post-hoc* test with Benjamini–Hochberg *p*-value correction. Value for *H*_0_ rejection was set at 0.05.

For bacterial communities, differences in overall diversity between diseased and healthy matrices, based on the Shannon Index, were statistically significant for rhizosphere and soil samples, whereas no significant differences were found in roots (Figure 2A). In both cases, healthy samples presented lower diversity compared to diseased samples. In addition, both rhizosphere and soil samples, regardless of health status, presented higher diversity values compared to their root equivalents (Figure 2A). Differences in the number of observed features were significant only between healthy and diseased rhizosphere samples, but not in soil and root samples (Figure 2B). Samples associated with healthy plants did not show statistically significant differences between matrices. However, rhizosphere samples associated with diseased plants contained a significantly higher number of observed features compared to both root and soil samples (Figure 2B). Finally, the evenness of bacterial ASV (Figure 2C) was tested with the Pielou index, which returned no significant difference between health status for roots, soil, and rhizosphere, although statistically significant differences were identified between root samples and non-root samples.

**Figure 3:**
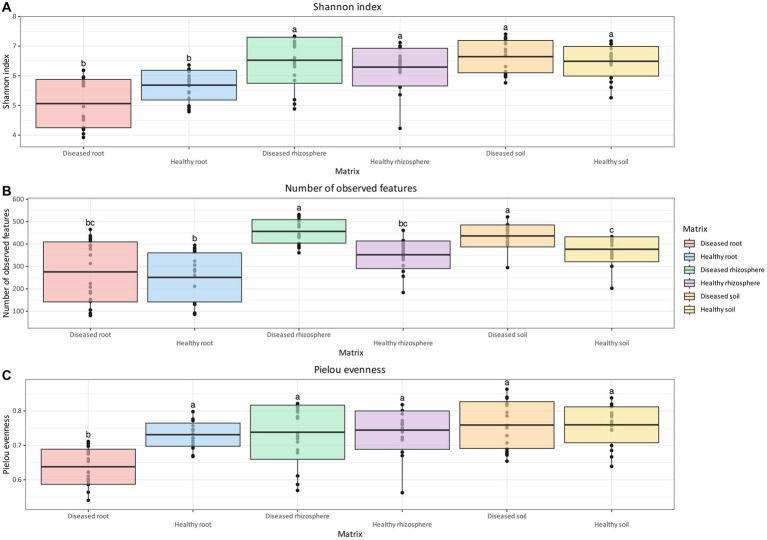
Box and whisker plot of Shannon Index **(A)**, number of observed features **(B)**, and Pielou evenness **(C)** measured across matrix and health status for fungi. Whiskers extend to ±1.5 interquartile range. The presence of statistically significant differences was assessed by means of a Kruskal–Wallis test, followed by a Dunn *post-hoc* test with Benjamini–Hochberg *p*-value correction. Value for *H*_0_ rejection was set at 0.05.

Unlike bacterial communities, the Shannon Index for fungal communities was not significantly different between health status for all considered matrices. However, similar to bacteria, both rhizosphere and soil samples had a higher diversity of fungi compared to root samples, regardless of health status (Figure 3A). The number of observed features (Figure 3B) in the fungal community of healthy plants was significantly lower than that of diseased plants in the rhizosphere and soil, but not in root samples. The number of observed features (Figure 3B) in the fungal communities of healthy plants was significantly lower than that of diseased plants in the rhizosphere and soil, but not in root samples. A significant reduction in the number of observed features was observed in healthy root tissue relative to healthy soil, but not in the rhizosphere. The number of observed features was also significantly lower in diseased roots relative to both diseased soil and rhizosphere samples. For Pielou evenness (Figure 3C), the only statistically significant difference was found between healthy and diseased root samples, with the former having higher values compared to the latter (*q*-value = 0.002).

**Figure 4:**
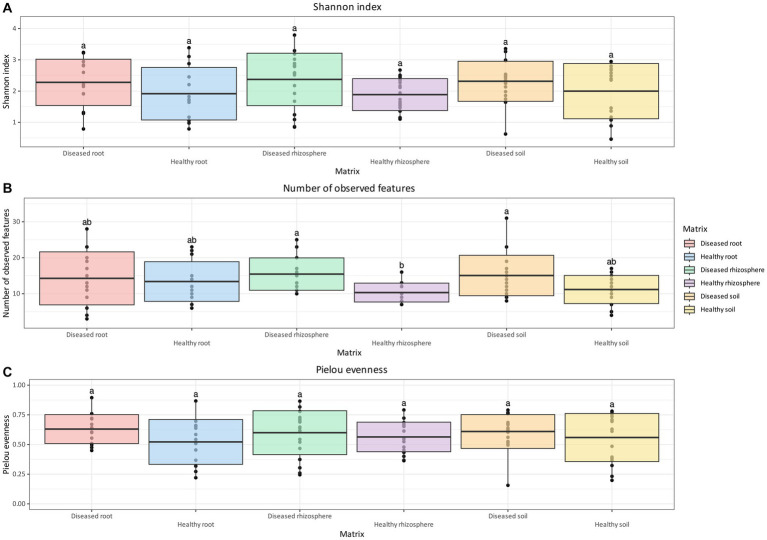
Box and whisker plot of Shannon Index **(A)**, number of observed features **(B)**, and Pielou evenness **(C)** measured across matrix and health status for oomycetes. Whiskers extend to ±1.5 interquartile range. The presence of statistically significant differences was assessed by means of a Kruskal–Wallis test, followed by a Dunn *post-hoc* test with Benjamini–Hochberg *p*-value correction. Value for *H*_0_ rejection was set at 0.05.

For oomycetes, no statistically significant differences were identified for either the Shannon Index or the Pielou Evenness (Figures 4A,C) between matrices and health status. However, like bacteria and fungi, the oomycete communities of diseased soil and rhizosphere were found to contain significantly more observed features than healthy rhizosphere (Figure 4B). In addition, diseased root communities were not significantly different in terms of observed features compared to the rhizosphere and soil, regardless of health status (Figure 4B).

The effect of sampling site on microbial community composition, along with disease status, matrix, and the interaction between these factors, was measured by means of a permutational analysis of variance (PERMANOVA) on robust Aitchison distances (Table 1).

**Table 1:** Results of multivariate permutational analysis of variance (PERMANOVA) on robust Aitchison distance matrix values for fungi, bacteria, and oomycetes, performed with package *adonis*.

Community	Effect	*F*.model	*R*^2^ (%)	Pr(>*F*)
Fungi	Disease status	16.97	0.7	0.001
	Matrix	109.77	8.9	0.001
	Site	554.74	67.9	0.001
	Disease status and matrix	9.55	0.8	0.001
	Disease status and site	92.64	11.3	0.001
	Matrix and site	14.42	3.5	0.001
	Disease status and matrix and site	11.87	2.9	0.001
	Residuals	NA	4	NA
Bacteria	Disease status	0.097	0	0.808
	Matrix	1837.34	86.9	0.001
	Site	96.19	7	0.001
	Disease status and matrix	29.84	1.4	0.001
	Disease status and site	4.46	0.3	0.001
	Matrix and site	11.28	1.6	0.001
	Disease status and matrix and site	4.49	0.6	0.001
	Residuals	NA	2.2	NA
Oomycetes	Disease status	24.83	9	0.001
	Matrix	34.26	24	0.001
	Site	12.33	13	0.001
	Disease status and matrix	1.03	0.6	0.386
	Disease status and site	13.67	14.2	0.001
	Matrix and site	3.82	8	0.001
	Disease status and matrix and site	1.98	2.7	0.06
	Residuals	NA	28.5	NA

Considered parameters include disease status, sampling matrix, sampling site, and their intersections. F.model is the *F*-statistic for the test, while *R*^2^ is the explained variance, expressed as percentage of the total variance. Pr(>*F*) is the false discovery rate (FDR) adjusted *p*-value.

**Figure 5:**
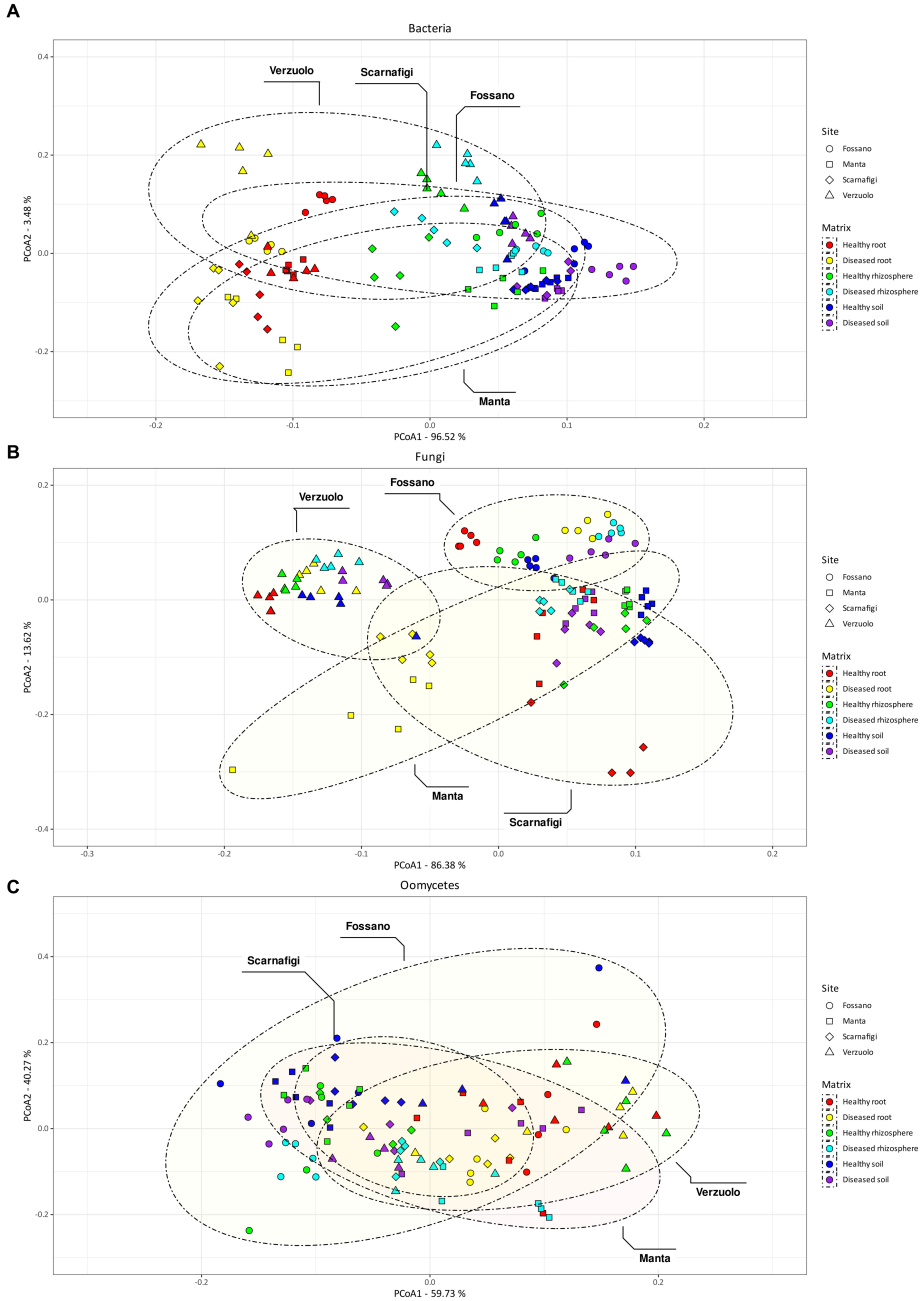
Non-metric multidimensional scaling (NMDS) plot of Aitchison distances between bacterial samples **(A)**, fungal samples **(B)**, and oomycetes **(C)**. Colors indicate different matrix/health status combination, while shape and ellipsoids are associated with the sampling site.

The obtained values of *R*^2^ indicated that the major effect on bacterial community composition was exerted by matrix, which accounted for 87% of the variance (Pr = 0.001; Table 1). Pairwise comparisons with PERMANOVA and PERMDISP between matrices (Table 2) identified significant differences in bacterial community composition between all matrix combinations; significant differences in dispersion were also identified. The effect of matrix on bacterial community composition was mirrored in the PCoA plot (Figure 5A), in which roots formed a distinct cluster from the soil and rhizosphere.

**Table 2:** Results of the permutational multiple analysis of variance (PERMANOVA) and multivariate dispersion analysis (PERMDISP) for bacteria, fungi, and oomycetes communities, with comparisons between matrices, sampling sites, and disease status.

	PERMANOVA		PERMDISP	
Comparison	Pseudo-*F*	*q*-value	*F*-value	*q*-value
Bacteria - matrix				
Rhizosphere—Root	335.16	0.001	2.85	0.048
Rhizosphere—Soil	52.41	0.001	10.47	0.004
Root—Soil	886.47	0.001	2.56	0.004
Bacteria - site				
Fossano—Manta	1.14	0.300	0.14	0.739
Fossano—Scarnafigi	5.02	0.132	0.13	0.739
Fossano—Verzuolo	3.88	0.132	0.52	0.739
Manta—Scarnafigi	2.12	0.217	0.61	0.739
Manta—Verzuolo	3.09	0.132	0.09	0.739
Scarnafigi—Verzuolo	1.59	0.258	1.67	0.739
Bacteria - disease status				
Healthy—Diseased	<0.01	0.990	1.40	0.237
Fungi—matrix				
Rhizosphere—Root	5.72	0.018	2.13	0.207
Rhizosphere—Soil	1.18	0.261	0.17	0.690
Root—Soil	9.86	0.003	4.23	0.123
Fungi—site				
Fossano—Manta	12.55	0.001	1.32	0.316
Fossano—Scarnafigi	25.53	0.001	5.86	0.034
Fossano—Verzuolo	314.69	0.001	4.47	0.016
Manta—Scarnafigi	0.99	0.344	0.23	0.657
Manta—Verzuolo	147.24	0.001	4.02	0.016
Scarnafigi—Verzuolo	192.12	0.001	13.30	0.006
Fungi—disease status				
Healthy—Diseased	0.82	0.385	1.29	0.261
Oomycetes—matrix				
Rhizosphere—Root	13.16	0.001	3.50	0.147
Rhizosphere—Soil	8.77	0.001	0.48	0.473
Root—Soil	31.00	0.001	1.43	0.319
Oomycetes—site				
Fossano—Manta	1.73	0.304	0.00	0.964
Fossano—Scarnafigi	0.50	0.620	8.88	0.009
Fossano—Verzuolo	14.36	0.003	1.57	0.205
Manta—Scarnafigi	0.64	0.570	14.45	0.006
Manta—Verzuolo	5.64	0.024	2.81	0.121
Scarnafigi—Verzuolo	14.48	0.003	8.97	0.012
Oomycetes—disease status				
Healthy—Diseased	9.68	0.001	6.68	0.011

Pseudo-*F* and *F*-value are the test metrics for PERMANOVA and PERMDISP, respectively. Q-value indicates the false discovery rate (FDR) adjusted *p*-value. Significance threshold for null hypothesis rejection was set at 0.05.

By comparison, the effect of site explained the largest amount of variance in fungal community composition, 68% (Pr = 0.001). Therefore, relative to bacterial communities, fungal communities were highly location-specific, with matrix having less of an effect (9%; Pr = 0.001). Pairwise comparisons between matrices identified statistically significant differences between root samples and rhizosphere/soil samples, with no statistically significant difference in dispersion. On the other hand, pairwise comparisons between sites identified statistically significant differences in fungal community composition between all sites (Fossano, Manta, Scarnafigi, and Verzuolo), except for Manta versus Scarnafigi. In addition, PERMDISP identified statistically significant differences in dispersion between sites. Fungal communities associated with Manta had comparable dispersion with those of Fossano and Scarnafigi (Table 2). This location effect on fungi was mirrored in the PCoA plot (Figure 5B), in which points associated with Verzuolo and Fossano formed distinct clusters, while points associated with Manta and Scarnafigi did not form distinct clusters.

Significant dissimilarities in oomycete communities were largely related to the matrix (24%; Pr = 0.001), site (13%; Pr = 0.001), and disease status (9%; Pr = 0.001). The statistical significance of the effect of disease status on oomycete community structure was confirmed by pairwise comparison, although a dispersion effect could not be ruled out. Additionally, pairwise comparisons indicated statistically significant differences in oomycete community composition between all matrices, with no significant differences in dispersion. Regarding site, pairwise PERMANOVA indicated a statistically significant difference in oomycete community composition between Verzuolo and all other locations, although a statistically significant difference in dispersion was found between Scarnafigi and all other geographical locations. The presence of multiple factors with relatively low effect size was mirrored by the PCoA plot (Figure 5C), in which no clear clustering patterns were identified.

### 3.3 Bacterial community composition

**Figure 6:**
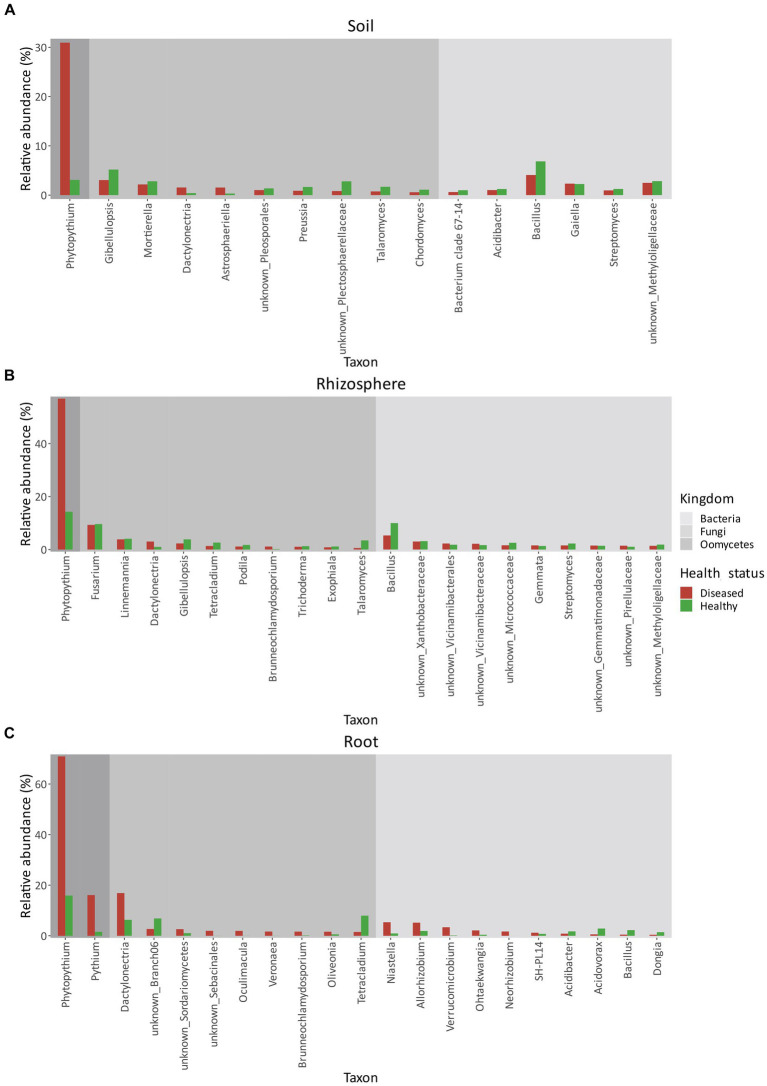
Relative abundances (%) of bacteria, fungi, and oomycetes in soil **(A)**, rhizosphere **(B)**, and root **(C)** between healthy and diseased orchards. Only taxa with a relative abundance higher than 1% in either condition were considered.

Bacterial communities across the three matrices—soil, rhizosphere, and root (Figures 6A–C)—did not show any dominant genera but contained a diversity of taxa, even when grouped by geographic location.

In terms of relative abundance (Figure 6A), *Bacillus* was the most abundant genus in both healthy (6.85%) and diseased (4.08%) orchard soil. In both healthy and diseased soil, all bacterial genera were similar in composition and abundance and included *Methyloligellaceae* (2–3%), *Gaiella* (~2%), *Streptomyces* (~1%), and *Acidibacter* (~1%).

As in soil, in the rhizosphere (Figure 6B), *Bacillus* was the most abundant bacterial genera. However, its relative abundance was roughly double in healthy plants (10.00%) relative to diseased plants (5.32%). No other significant differences in bacterial relative abundance were identified between healthy and diseased plants. Bacterial genera identified also included *Xanthobacteriaceae* (~3%), *Vicinamibacteriales* (~2%), *Vicinamibacteraceae* (~1–2%), *Micrococcaceae* (~1–2%), *Gemmata* (~1–2%), *Streptomyces* (~1–2%), *Gemmatimonadaceae* (~%1–2%), *Pirellulaceae* (~1%), and *Methyloligellaceae* (~1–2%). In healthy root tissues (Figure 6C), the most abundant genus was *Acidovorax* (2.87%), followed by *Bacillus* (2.25%), *Allorhizobium* (1.91%), *Acidibacter* (1.74%), and *Dongia* (1.43%). Other genera, including *Niastella*, *Ohtaekwangia*, *Verrucomicrobium*, *Neorhizobium*, and SH-PL14, represented approximately 2% of the root community composition. In KVDS-affected roots (Figure 6C), the most abundant genera were *Niastella* (5.36%) and *Allorhizobium* (5.19%), followed by *Verrucomicrobium* (3.38%), *Ohtaekwangia* (2.12%), *Neorhizobium* (1.70%), and SH-PL14 (1.22%), while the remaining 2.29% of the root bacterial community was made up of *Acidibacter*, *Acidovorax*, *Bacillus*, and *Dongia*.

### 3.4 Fungal community composition

Like bacteria, fungal communities also contained a wide diversity of genera in the three matrices (Figures 6A–C).

The most abundant fungal genera in soil (Figure 6A) were *Gibelluliopsis* in both healthy (5.19%) and diseased (3.05%) orchards, followed by *Mortierella* spp. both in healthy (2.81%) and diseased (2.17%) orchards. Other major genera composing the fungal community in diseased soil were: *Dactylonectria* (1.55%), *Astrosphaeriella* (1.54%), *Pleosporales* (1.02%), followed by the less abundant *Preussia* (0.88%), *Plectosphaerellaceae* (0.82%), *Talaromyces* (0.75%), and *Cordomyces* (0.58%). The fungal community composition was similar in healthy soils, with minor differences in relative abundance: *Plectosphaerellaceae* (2.80%), *Talaromyces* (1.69%), *Preussia* (1.66%), *Pleosporales* (1.37%), *Chordomyces* (1.10%), *Dactylonectria* (0.40%), and *Astrosphaeriella* (0.32%).

In the rhizosphere (Figure 6B), the predominant genus was *Fusarium* (9.31%) in diseased sites, followed by *Linnemania* (3.85%), *Dactylonectria* (3.02%), *Gibellulopsis* (2.31%), *Tetracladium* (1.34%), *Podila* (1.14%), *Brunneochlamydosporium* (1.13%), *Trichoderma* (1.04%), *Exophiala* (0.85%), and *Talaromyces* (0.57%). As in soil, differences between fungal genera in healthy versus diseased rhizosphere were small (Figure 6B).

In roots, however (Figure 6C), *Dactylonectria* was highly present in KVDS-affected tissues (16.89%), followed by unknown Branch06 (2.70%), *Sordariomycetes* (2.62%), *Sebacinales* (1.97%), *Oculimacula* (1.94%), *Veronaea* (1.68%), *Brunneochlamydosporium* (1.65%), *Oliveonia* (1.61%), and *Tetracladium* (1.52%). In healthy tissues, *Dactylonectria* was less predominant (6.31%), while *Tetracladium* (7.96%) was the most abundant fungal genera, followed by unknown Branch06 (6.86%). Less abundant genera included *Sordariomycetes* (1.05%) and *Oliveonia* (0.58%), while *Sebacinales*, *Oculimacula*, *Veronaea*, and *Brunneochlamydosporium* accounted for <1%.

### 3.5 Oomycete community composition

**Figure 7:**
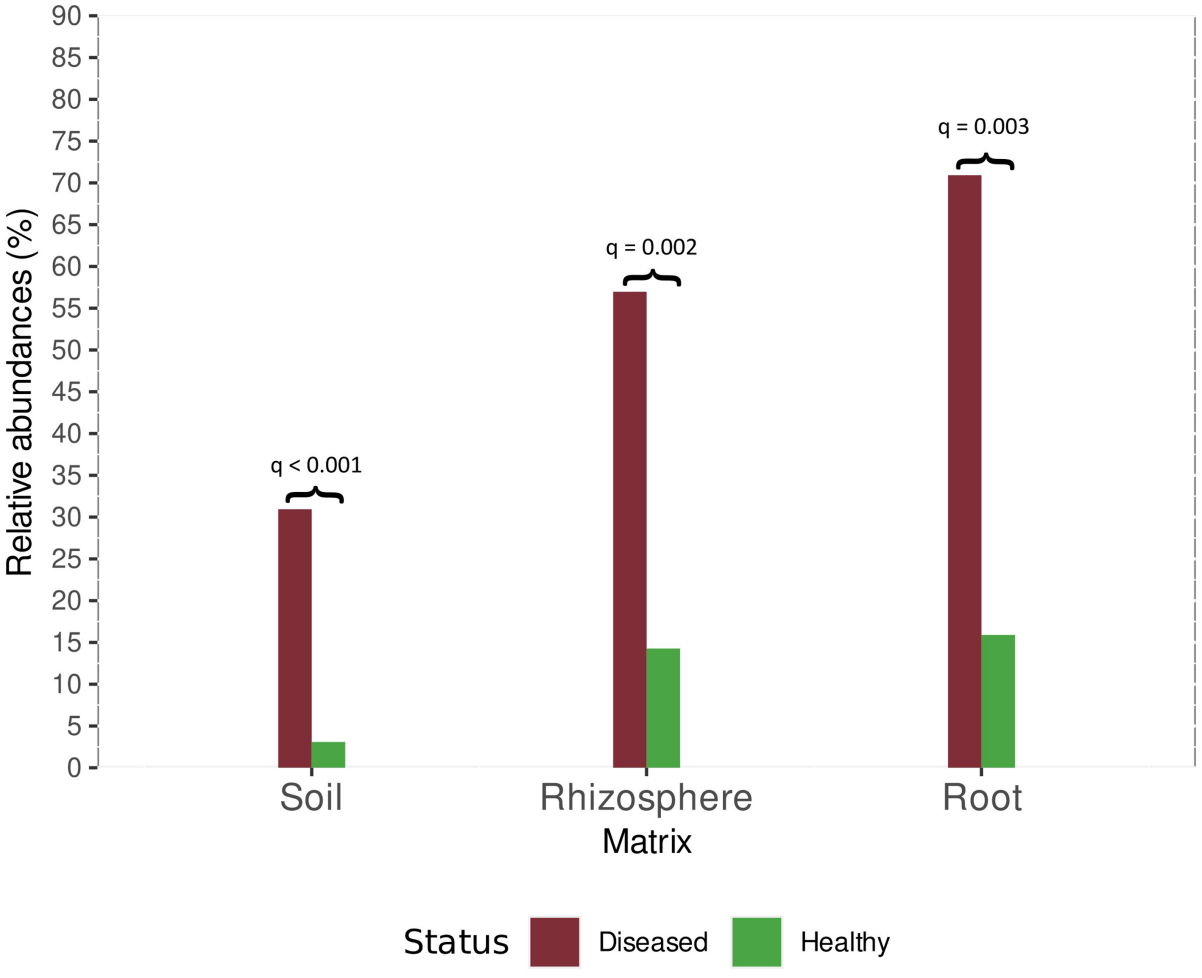
Relative abundance of *Phytopythium* in healthy and diseased plants for root, rhizosphere, and soil samples. For each comparison, the Bonferroni-adjusted *p*-value is provided, as returned by the ALDEx2 analysis.

Among oomycetes detected in soil, a significant difference in the relative abundance of *Phytopythium* spp. was observed between healthy and diseased sites. In samples collected from diseased orchards, the abundance of *Phytopythium* was as high as 30.94%. In healthy soil, the relative abundance was 3.09% (Figure 7).

The same trend was observed in the rhizosphere, where *Phytopythium* relative abundance was 56.97 and 14.27% of the total oomycete community at diseased sites and healthy sites, respectively (Figure 7).

In comparison, *Phytopythium* relative abundance reached 70.92% in diseased roots and 15.90% in healthy roots (Figure 7). *Pythium* spp., another KVDS-associated genus, was also detected in the roots of healthy (1.57%) and affected (16.10%) plants (Figure 6C). In soil (Figure 6A) and rhizosphere (Figure 6B), *Pythium* spp. did not reach a frequency above 1% and were therefore not listed in Figure 6.

### 3.6 Association networks among microbial taxa

Cross-kingdom co-occurrence networks based on relative abundance profiles among fungi, bacteria, and oomycetes were investigated with CoNet (Supplementary Figure S1). In all networks, bacteria represented the most abundant kingdom, followed by fungi (Table 3). Other taxa were far less abundant, with oomycetes having a relatively higher presence in the rhizosphere compared to root and soil. Comparison between healthy and diseased plants (Table 3) indicated a reduction of co-presence connections (indicating positive associations among taxa) and an increase in co-exclusion connections (indicating negative associations among taxa) in diseased rhizosphere and soil, while the opposite was true for root samples. It is also notable that in diseased (relative to healthy) root tissue, there was an increase in the relative abundance of oomycete nodes, the average number of neighbors, network heterogeneity, network centralization, and the number of connected components. Taken together, these results suggest that the microbial endophytic network becomes increasingly unequal due to the disease condition (Table 3). In soil, the decrease in connected components was associated with a reduction in both the number of nodes and edges, while in the rhizosphere, this was associated with an increase in both metrics. The average number of neighbors was higher for healthy samples compared to diseased samples in root and soil and slightly lower in the rhizosphere, while the network radius was higher for healthy samples in soil, lower in the rhizosphere, and did not change in roots.

**Table 3:** Network metrics and parameters for root, rhizosphere, and soil microbiota.

	Root		Rhizosphere		Soil	
	Healthy	Diseased	Healthy	Diseased	Healthy	Diseased
Bacteria	69.47	69.17	62.11	64.25	62.35	63.58
Fungi	29.42	29.09	36.11	34.2	36.28	35.43
Oomycetes	0.07	0.37	1.11	0.6	0.12	0.2
Rhizaria	0.53	0.43	0.48	0.43	0.06	0.07
Archaea	0	0	0	0.33	0.37	0.2
Ichthyosporia	0	0	0.24	0	0.25	0
Unidentified	0.53	1.33	0.36	0.6	0.56	0.52
Co-presence	54.21	59.19	54.39	51.6	53.04	48.59
Co-exclusion	45.79	40.81	45.61	48.4	46.96	51.41
Number of nodes	1,523	1,612	1,681	1845	1,615	1,524
Number of edges	8,893	8,030	9,426	10,992	8,953	8,165
Avg. number of neighbors	14.03	19.21	12.15	22.53	14.01	11.5
Network diameter	13	13	12	13	16	12
Network radius	7	7	7	7	8	6
Characteristic path length	8.49	10.53	10.21	8.24	9.13	16.13
Clustering coefficient	2.54	0.19	0.18	3.04	3.03	2.59
Network density	0.01	0.08	0.08	0.07	0.08	0.08
Network heterogeneity	1.28	7.03	4.34	2.57	5.54	7.33
Network centralization	1.07	1.26	1.43	0.59	1.18	2.41
Number of connected components	126	149	88	66	100	60

For each matrix and status, the relative abundance of nodes (for fungi, bacteria, and oomycetes) in each network is reported. In addition, the relative abundance of co-presence/co-exclusion nodes is indicated. Average number of neighbors: average number of nodes connected to each node; network diameter: value of the highest eccentricity of the graph; network radius: length of the lowest eccentricity of the graph; characteristic path length: average path length of the graph; clustering coefficient: average of the clustering coefficient of the graph nodes, defined as the ratio between the number of edges connecting the neighbor nodes and the maximum theoretical number of edges connecting the neighbor nodes; network density: ratio between the number of edges and the theoretical maximum number of edges in the graph; network heterogeneity: measure of the tendency of a graph to present hub nodes, which are nodes with a high number of edges; network centralization: measure of the tendency of the graph to have a single hub node; number of connected components: indicates the number of so-called “connected components,” which are subgraphs where each node has a pairwise connection to every other node.

**Figure 8:**
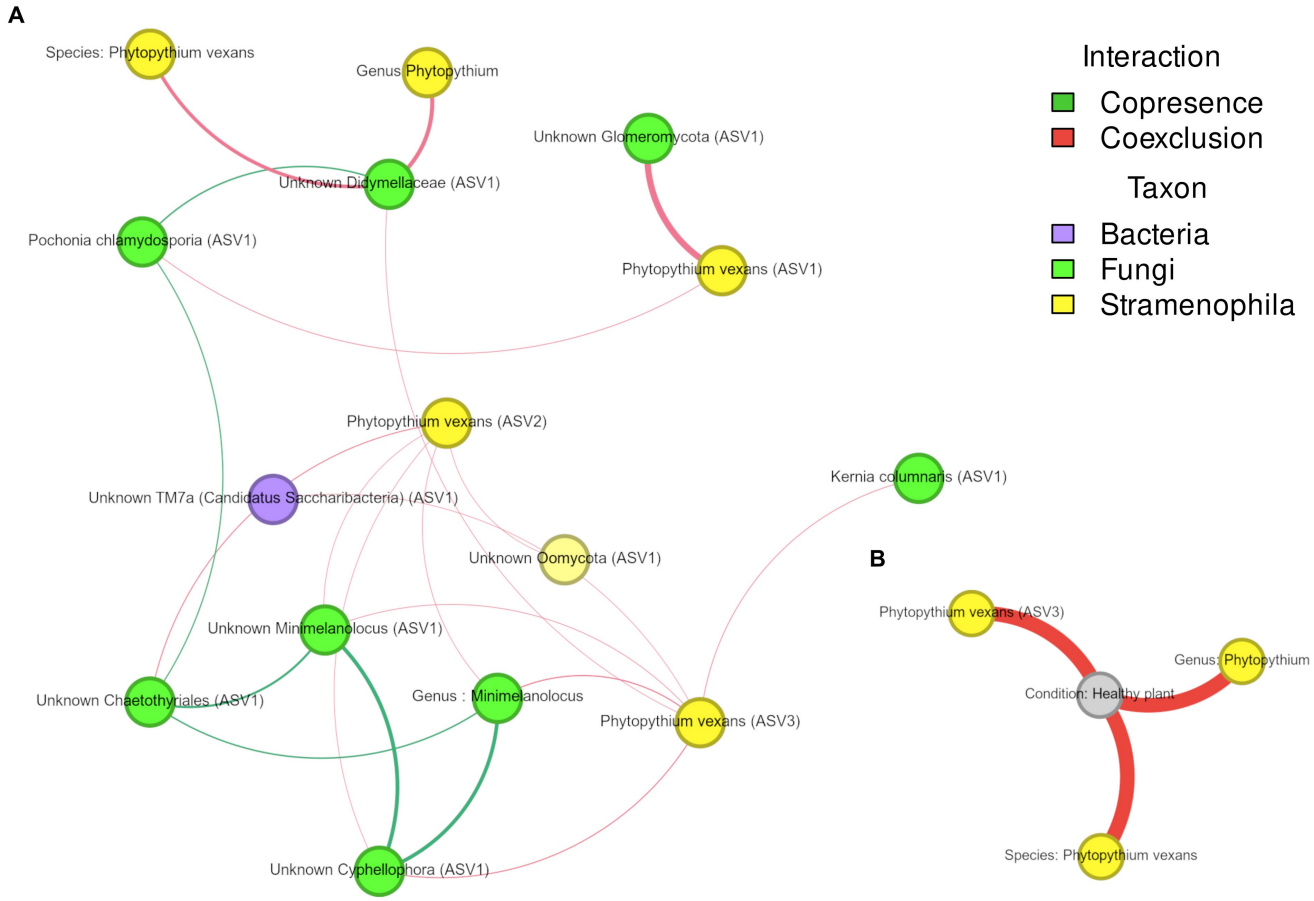
**(A,B)** Association network of *Phytopythium* in microbial communities associated with kiwi plant rhizosphere **(A)** and soil **(B)**. Edge color represents association type (co-presence of co-exclusion), whereas node color refers to their taxonomic kingdom. Node size is proportional to the number of connections (i.e., degree).

For *Phytopythium*, statistically significant associations were identified in the soil and rhizosphere, but not in the root (Figure 8). In the rhizosphere (Figure 8A), *P. vexans* ASV1 was found to be negatively correlated with ASVs belonging to *Pochonia chlamydosporia* and an unknown *Glomeromycota*; *P. vexans* ASV2 was also negatively correlated with ASVs belonging to the genera *Minimelanolocus* and *Cyphellophora*, as well as unknown organisms in the *Chaetothyriales* order and Oomycota class, but also with the genus *Minimelanolocus*. Finally, *P. vexans* ASV3 was negatively correlated with an ASV of species *Kernia columnaris*, ASVs belonging to the genera *Minimelanolocus* and *Cyphellophora*, as well as unknown organisms in the *Didymellaceae* family and TM7a phylum, and with the genus *Minimelanolocus*. In addition, both species of *P. vexans*, as well as an additional ASV belonging to the genus *Phytopythium*, were negatively correlated with an ASV associated with unknown *Didymellaceae*. In soil (Figure 8B), *Phytopythium* (as genus, as the species *P. vexans*, and as *P. vexans* ASV3) was negatively correlated with the healthy status.

### 3.7 Evaluation of rhizosphere ecological processes

The impact of stochastic and deterministic processes on microbial composition was extrapolated via comparative analysis of beta Nearest Taxon Index (bNTI) values across different samples. In particular, bNTI values higher than 2 or lower than −2 can be considered the result of prevailing deterministic processes (i.e., selection), while values between −2 and 2 are associated with a predominance of stochastic processes (Stegen et al., 2013). Fungal communities had a lower average percentage of turnover explained by selection (1.84%) than bacteria (12.63%) and oomycetes (29.47%). When the health status of these communities was considered, differences emerged between taxa. For fungal and oomycetes communities, selection had a greater impact on the turnover of diseased communities (2.62 and 43.16%, respectively) than on healthy communities (1.05 and 17.54%, respectively). In contrast, for bacterial communities, there was a higher impact of selection on the turnover of healthy communities (16.31%) than diseased communities (8.94%). Despite these differences among taxa, stochastic processes represented the main driver of community turnover for all taxa, with an impact of 98.16, 87.37, and 70.53% for fungal, bacterial, and oomycete communities, respectively. Stochastic processes can be additionally classified according to Bray–Curtis-derived Raup-Crick (RCbray) values. RCbray < −0.95 is considered the result of homogenizing dispersal (a combination of drift and high dispersal); RCbray >0.95 is considered the result of dispersal limitation (a combination of drift and low dispersal); and −0.95< RCbray <0.95 is considered the result of ecological drift (drift acting alone; Stegen et al., 2013). For fungi and bacteria (Supplementary Figures S2, S3), dispersal limitation was the main stochastic process both in healthy communities (80.00 and 78.61%, respectively) and diseased communities (80.92 and 76.10%, respectively). For healthy fungal communities, ecological drift was higher (12.23%) than homogenizing dispersal (7.44%), while in diseased communities, the opposite (8.10% vs. 11.89%, respectively) was predicted. For bacterial communities, homogenizing dispersal was similarly high in healthy communities (16.76%) and diseased communities (16.89%), while ecological drift was marginal for both kinds of communities (2.31 and 6.92%, respectively). For oomycetes (Supplementary Figure S4), no dispersal limitation was observed, and homogenizing dispersal was measured only in diseased communities (6.48%). In contrast to both fungi and bacteria, oomycete communities were predicted to have high values of ecological drift in both diseased communities (93.52%) and healthy communities (100.00%).

### 3.8 Physicochemical characteristics of soils and correlation with biotic components

**Figure 9:**
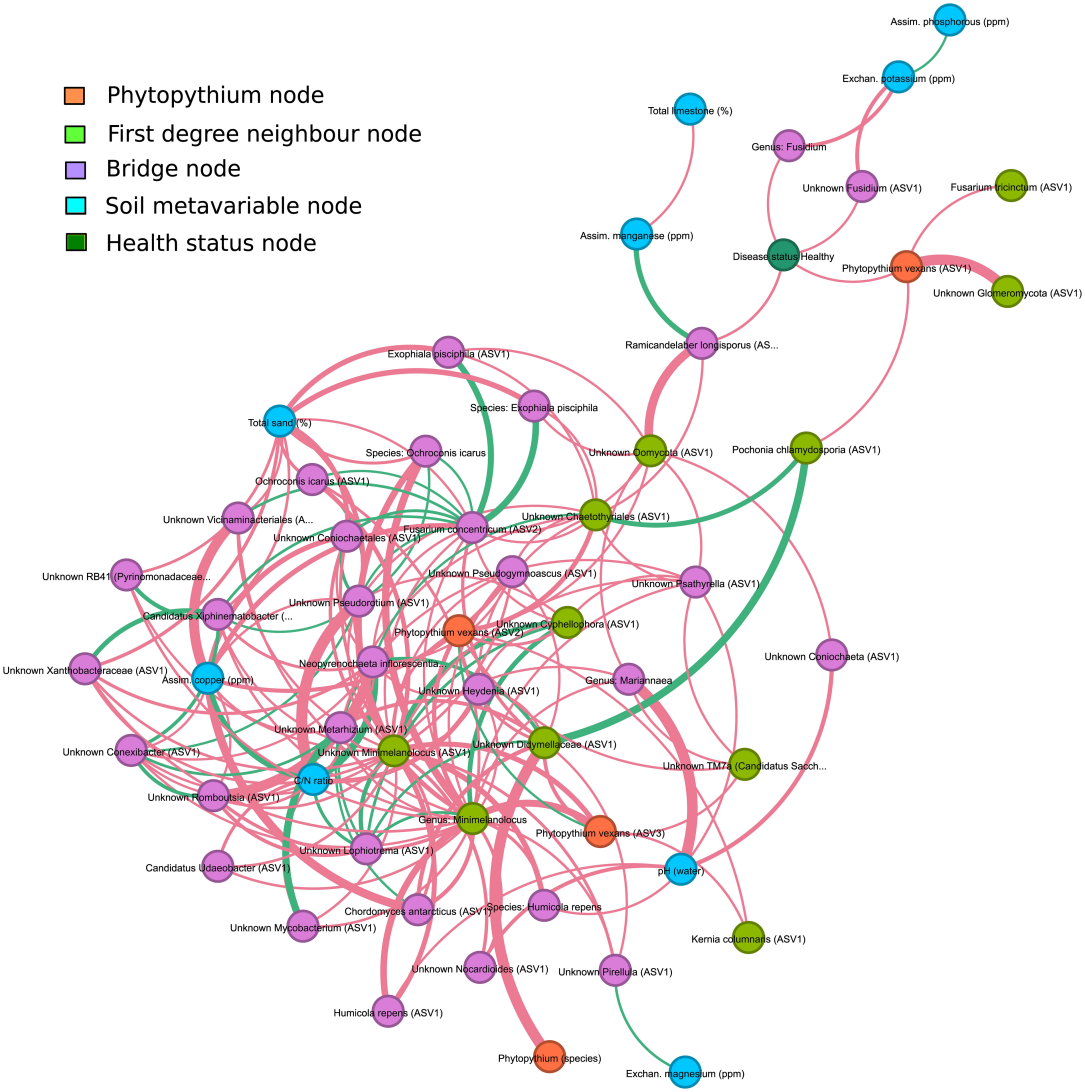
Interaction network of the rhizosphere microbiome and soil meta-variables, filtered to underline interactions between *Phytopythium*, interacting taxa and meta-variables. Orange nodes are associated with *Phytopythium*; light green nodes are the first-degree neighbor nodes of *Phytopythium*, i.e., have direct interaction with *Phytopythium*; blue nodes are associated with soil meta-variables; dark green nodes are associated with the health status of communities; and finally, the purple nodes are bridge nodes, which are defined as nodes sitting on the shortest path between *Phytopythium* and soil meta-variables. Edges are colored based on interaction, which can be either co-presence (green) or co-exclusion (red). Edge thickness is directly proportional to the strength of interaction.

Chemical–physical analysis of the sampled soils considered different variables as soil composition in terms of physical properties and the presence/exchange of chemical elements (Supplementary Table S3). NMDS analysis of these data indicated that healthy and diseased soils did not cluster separately (Supplementary Figure S5). This result suggests the absence of chemical/physical factors strictly associated with disease status and/or microbiome assembly. Soil physicochemical properties have been reported as important drivers of soil microbial communities, which represent the main reservoir for the rhizosphere microbiome (Plassart et al., 2019). Therefore, these data were integrated with the rhizosphere network (Figure 9).

Associations with microbial taxa were considered for assimilated copper (ppm), assimilated manganese (ppm), assimilated phosphorous (ppm), total sand (%), total limestone (%), C/N ratio, exchanged magnesium (ppm), exchanged potassium (ppm), and water pH. Although these parameters did not show any direct interaction with the main oomycete, *Phytopythium* spp., third-degree (or higher) associations with the genera interacting with *Phytopythium* were identified in the rhizosphere (Figure 9). Health status was also included as a parameter and resulted in third-degree associations with exchanged potassium and assimilated manganese. Assimilated manganese showed a positive association with *Ramicandelaber longisporus*, which was also negatively associated with the healthy status of the plant, which is, in turn, negatively linked to *P. vexans* (ASV1). Exchanged potassium was negatively associated with the genus *Fusidium* and *Fusidium* (ASV1), which were negatively correlated with the healthy status of the plant. At a higher degree of association, compared to *Phytopythium* or the healthy status, exchanged potassium is positively correlated with assimilated phosphorus, while assimilated manganese is negatively associated with total limestone. Exchanged magnesium was positively associated with an unknown *Pirellula* ASV1, which was negatively associated with *P. vexans* ASV3. The C/N ratio was negatively correlated with an unknown *Minimelanolocus* ASV1, which was negatively correlated with *P. vexans* ASV3. Other parameters, including assimilated copper and total sand, were associated (at higher degrees) in the neighborhood with both the healthy status of the plant and *P. vexans* ASVs, making their effects difficult to predict.

### 3.9 Quantification of *Phytopythium vexans* across matrices via qPCR and isolation from plant roots

Relative quantification of the oomycete *P. vexans* in soil, rhizosphere, and roots was obtained by generating a standard curve (*R*^2^ = 0.994, E = 95%) from seven 10-fold dilutions (ranging from 20 fg to 20 ng) of *P. vexans* genomic DNA (strain CBS 119.80). The quantification of the target was calculated using linear regression with the equation associated with the curve *y* = −3.4578*x* + 20.465. The results are listed in Supplementary Table S4. Isolates of *P. vexans* were constantly reisolated from diseased plant roots (sampled from diseased orchards) and identified by using macro- and micromorphological analysis and sequencing, as described in Prencipe et al. (2023). Five representative isolates were deposited in the Turin University Culture Collection (TUCC; Supplementary Table S5).

## 4 Discussion

So far, most of the research on KVDS has been focused on defining the causal agents of the disease using traditional and omic-based techniques. This body of research has revealed the involvement of multiple species of oomycetes belonging to the genera *Phytopythium* and *Phytophthora* in the onset of the syndrome in different producing areas of Italy. In this study, we not only characterized the bacterial, fungal, and oomycete communities present in eight different orchard locations in Cuneo province (four healthy and four affected sites), but we also explored microbial community dynamics across three different belowground compartments. In addition, cross-kingdom microbial networks were constructed to explore potential interactions between taxa and address the possibility of dysbiosis. Potential associations between microbial taxa and soil-associated abiotic factors were also explored. The finding that *Phytopythium* spp. were present both in healthy and diseased orchards suggests that the onset of KVDS is likely related to a shift in the larger microbial community rather than being solely due to these oomycetes. In this study, *Phytophthora* spp. were not identified as being abundant in any sample type (<1% relative abundance). When all locations were considered, oomycetes were strongly correlated with disease status compared to fungi and bacteria. More specifically, relative abundances of *Phytopythium* spp. were significantly higher in all diseased matrices compared to healthy ones. However, no significant differences in oomycetes alpha diversity metrics were identified between matrices, except that the healthy rhizosphere had a reduced number of observed features relative to the diseased rhizosphere. This trend was also observed for fungal and bacterial communities and may suggest a dramatic shift in the way microbial communities interact with each other and with the host (Somera and Mazzola, 2022).

Previous studies conducted in Northeast Italy by Savian et al. (2022) characterized KVDS-associated communities of kiwi root endosphere and rhizosphere using a metabarcoding approach. Compared to our study, this analysis only considered one healthy site and three sampling points from a single diseased orchard. Savian et al. (2022) identified *P. vexans* in both diseased and healthy root endosphere and rhizosphere. In addition, *P. sojae* was related to KVDS onset, though its pathogenicity on *Actinidia deliciosa* has not yet been verified by reproduction of KVDS symptoms after inoculation. The involvement of the *Phytopythium* genus in KVDS was also reported in a previous study by Savian et al. (2020) in which isolation from roots was performed, resulting in a high occurrence of Pythiaceae. Most notably, *P. chamaehyphon* and *P. vexans* were reported to be associated with the syndrome. In another study performed on root tissues of kiwi vines sampled in northern Italy, the oomycete genera *Phytophthora* and *Phytopythium* were isolated from both health statuses, with a higher percentage in diseased plants (Donati et al., 2020). The presence of the same oomycete genera (*Phytophthora* spp., *Phytopythium* spp., and *Pythium* spp.) was also observed in both diseased and healthy soybean rhizospheres (Noel et al., 2020). This finding suggests that a quantitative imbalance of species may contribute to symptom development rather than simply the presence or absence of highly pathogenic species.

*Phytopythium* spp. pathogenicity on kiwifruit and the ability to reproduce symptoms in controlled conditions have been verified by different authors. Prencipe et al. (2020) tested the pathogenicity of *P. vexans* on kiwifruit plantlets and reproduced KVDS symptoms, fulfilling Koch postulates. Savian et al. (2021) verified the pathogenicity of another species, *P. chamaehyphon*, on *Actinidia deliciosa* cv. Hayward and observed the typical wilting symptoms with disease progression similar to field conditions. A previous study in Northwestern Italy, which included the orchards considered in this study, revealed a high occurrence of *Phytopythium* spp. isolated from the root tissues of kiwi plants showing clear symptoms of KVDS (Prencipe et al., 2023). These findings complement the metabarcoding results of this study.

Analyses of fungal communities associated with KVDS in healthy and diseased samples were also explored. Fungal alpha diversity did not show any statistically significant difference in the Shannon Index comparing healthy and diseased matrices. However, root tissue diversity (in terms of observed features) was lower than that of soil and the rhizosphere. This observed reduction is expected due to the numerous factors differentiating root communities from the surrounding compartments (Bulgarelli et al., 2013). This finding was also observed in previous studies conducted in forest ecosystems (Frey et al., 2021). In addition, fungal evenness was significantly lower only in diseased roots, as a combination of the presence of a prevalent pathogenic taxon and the effect of continuous cropping as previously observed in *Panax notoginseng* gardens (Tan et al., 2017).

In this study, *Fusarium* was the main fungal genus in the rhizosphere, regardless of disease status, whereas *Linnemania* and *Dactylonectria* characterized diseased samples. Although *Dactylonectria* was the main genus in affected roots, it was also present in healthy roots in lower abundance. These findings are consistent with Savian et al. (2022), who found a high abundance of Nectriaceae in both root tissue and rhizosphere, regardless of KVDS. Our findings on fungal community composition in kiwifruit orchard soils are consistent with previous works. *Dactylonectria* (or *Cylindrocarpon*-like species) was isolated from kiwifruit orchard soils and is already associated with kiwifruit cultivation (Tacconi et al., 2015; Manici et al., 2022). *Cylindrocarpon* spp. pathogenicity on kiwifruit was verified in a previous study, where symptoms of root rot were reproduced 60 days after inoculation (Erper et al., 2013). Savian et al. (2020) isolated Hypocreaceae, Ceratobasidiaceae, and Nectriaceae from KVDS-affected roots, with a high occurrence of *F. solani*. *Fusarium* spp. were recently reported as root rot agents on kiwifruit cultivated in poorly drained soils in China (Song et al., 2023), but the ability to reproduce KVDS symptoms was not directly verified. In previous studies conducted on apple replant disease (ARD), which affects apple trees worldwide, both Nectriaceae and *Fusarium* spp. were reported as fungal components of the biotic complex associated with the disease (Franke-Whittle et al., 2015). *Fusarium* spp. were frequently isolated from apple orchards but resulted in being non-pathogenic or weakly virulent when inoculated on plantlets, depending on the species (Tewoldemedhin et al., 2011a). Pathogenicity of *Cylindrocarpon* spp. on apples was tested in a previous study, and virulence resulted strain-dependent (Tewoldemedhin et al., 2010). Recent studies conducted on secondary metabolites produced by *Fusarium* spp. and Nectriaceae (*Cylindrocarpon*-like) showed the ability of these filamentous fungi, frequently isolated from multifactorial pathosystems, to significantly impact fruit tree growth by releasing phytotoxic compounds in soil (Manici et al., 2017, 2021). Based on these findings, the roles of *Fusarium* spp. and *Cylindrocarpon* spp. in KVDS need further investigation.

In regard to bacterial abundance, a wide variety of genera were identified but were not correlated with the health status of the orchard; instead, they were matrix-specific. Significant differences in bacterial community composition were identified in the rhizosphere and soil between healthy and diseased orchards. In both soil and rhizosphere matrices, *Bacillus* was the most prevalent genera, but we were not able to identify species-level differences characterizing KVDS-affected orchards. Bacterial alpha diversity metrics, in particular the Shannon Index and number of observed features, were higher in diseased samples of soil and rhizosphere (but not root) compared to healthy ones. Previous results highlighted the presence of higher bacterial diversity in samples associated with different microhabitats of diseased plants (Hossain et al., 2021; Obieze et al., 2023). In addition, root samples had a lower richness (in terms of observed features) compared to both the rhizosphere and soil, which also aligned with previous observations of bacterial populations in plants (Bulgarelli et al., 2012; Lundberg et al., 2012; Mukhtar et al., 2021; Yuan et al., 2022). Only a few studies on the biotic factors involved in KVDS onset have considered the bacterial microbiota. For example, Donati et al. (2020) characterized the bacterial communities in root samples from three kiwifruit orchards with different KVDS severity. In contrast to our findings, the bacterial communities of healthy roots were characterized by higher biodiversity than KVDS-affected roots. In another study, the soil bacterial composition of different kiwifruit orchards was characterized using NGS techniques (Manici et al., 2022). A shift toward nitrifying bacterial genera in soil samples from old orchards, showing typical decline symptoms together with a strong reduction of the overall bacterial community, was observed.

The results of beta-diversity analyses from this study confirm the large impact of the belowground compartment in defining the composition of bacterial communities, while geographical location had by far the highest impact on fungal community composition. The impact of the microhabitat on bacterial communities was previously investigated and was shown to be the main parameter behind beta-diversity variation (Bulgarelli et al., 2015; Jin et al., 2017; Yuan et al., 2018). On the other hand, soil physico-chemistry and site-specific soil properties, rather than host effects, are reported to strongly drive fungal assemblages (Adamo et al., 2021).

For oomycetes, statistically significant differences in observed features between healthy and diseased sites were observed, except for root tissue, with a greater number of ASVs associated with diseased conditions. However, neither matrix nor sampling location explained a significant part of the composition variance in the oomycete beta diversity. A study conducted in the soybean rhizosphere showed a similar increase in richness associated with the diseased condition and a statistically significant effect of the sampling site on community composition (Noel et al., 2020).

In ARD, no single biological organism was identified as a causal agent, leading to ambiguity in defining the determining factors. In the ARD pathosystem, species belonging to the genera *Rhizoctonia*, *Phytophthora*, *Cylindrocarpon*, *Pythium*, and *Phytopythium* have been identified as causal agents (Tewoldemedhin et al., 2011b; Mazzola and Manici, 2012; Tilston et al., 2018), presenting many resemblances with KVDS. Another woody plant decline system that presents similarities with KVDS is peach replant disease, in which *P. vexans* dominates the diseased community (Yang et al., 2012). This oomycete species was frequently identified both by culture-dependent and -independent methods, correlating with disease development, but also varied in abundance, which was attributed to interactions with abiotic factors. In addition, *F. solani* was also associated with the community of peach replant disease, similar to our findings in kiwifruit.

Co-exclusion/co-occurrence networks can help to provide a better picture of the structure of microbial communities. In general, no difference was found in the taxon composition of nodes across different microhabitats and health statuses of orchards in this study. Bacteria were the most represented kingdom, followed by fungi. In diseased soil, the reduction of both the number of edges and nodes, together with a reduction in the number of connected components and the average number of neighbors, suggests a decrease in interaction within the microbial network. In contrast, in the diseased rhizosphere, an increase in edge and node numbers was observed, with a slight increase in the average number of neighbors. This, combined with a decrease in connected components, may indicate a reduction in associations between pre-existing components and the appearance of new associations in diseased plants. Finally, in the network associated with diseased roots, an increase in the number of nodes and a decrease in the number of edges were observed relative to healthy roots. This was also associated with a decrease in the average number of neighbors and an increase in connected components. As in the rhizosphere, these data suggest a decrease in connections inside connected components with the formation/appearance of new ones.

A particular focus was given to associations between *Phytopythium* and other taxa, both in the soil and rhizosphere. In the root network analysis, however, *Phytopythium* was not significantly correlated with other taxa. Among detected *Phytopythium* ASVs, *P. vexans* ASV3 was the only taxon with a direct negative correlation with healthy soil, suggesting a predominant role of *P. vexans* in KVDS. In the diseased rhizosphere, this ASV was negatively correlated with multiple ASVs of Chaetothyriales (black yeasts), which includes the families Herpotrichiellaceae and Cyphellophoraceae (Teixeira et al., 2017). The family Cyphellophoraceae includes both the *Cyphellophora* and *Phialophora* genera. *Cyphellophora* is a fungal genus with several plant-associated species and is characterized by tolerance to high temperatures (Feng et al., 2014). The family Herpotrichiellaceae is largely composed of saprobic species and includes *Minimelanolocus*, a freshwater lignicolous fungal genus associated with decaying submerged wood debris (Shearer et al., 2007; Wang et al., 2019). The genus *Minimelanolocus* and *Minimelanolocus* ASV1 were both predicted to be co-exclusive with *P. vexans* ASV2 and ASV3. Similarly to the oomycetes associated with KVDS (Prencipe et al., 2023), this group of fungi is characterized by optimal growth at high temperatures (Hyde et al., 2016). In addition, *Cyphellophora* ASV1 and *Chaetothyriales* ASV1 were co-exclusive with *P. vexans* ASV2. *P. vexans* ASV3 was also co-exclusive with the fungus *Kernia columnaris* ASV1 (homotypic synonym: *Cephalotrichum columnare*), a soil saprobe with a worldwide distribution (Sandoval-Denis et al., 2016). *P. vexans* ASV2 was also negatively correlated with the ubiquitous bacterial phylum *Candidatus saccharibacteria* ASV1 (formerly Candidate Division TM7), which has been commonly found in soils, sediments, and wastewater (Ferrari et al., 2014). The observed co-exclusive associations of saprobes and saccharibacteria with *P. vexans* ASVs in the rhizosphere may imply a cross-kingdom competition for plant-derived carbon. Saccharibacteria can digest the plant cellular wall and ferment soil necromass and root exudates (Starr et al., 2018), therefore competing for nutrients. Negative correlations were also found between *P. vexans* ASV1 and beneficial organisms like Unknown *Glomeromycota* ASV1 and *Pochonia chlamydosporia* ASV1. *Glomeromycota* spp. are obligate biotrophs that have been reported to form arbuscular mycorrhizas (AMs) with kiwifruit ‘Hayward’ (Zhang et al., 2017). The nematophagous endophyte *Pochonia chlamydosporia* has been shown to promote root growth in tomato and banana (Zavala-Gonzalez et al., 2015; Mingot-Ureta et al., 2020). Finally, *P. vexans* was predicted to be co-exclusive with Didymellaceae ASV1. Didymellaceae is a fungal family rich in species inhabiting different ecosystems and has been reported to associate with a broad range of plant hosts, including *Actinidia* spp. Most species of Didymellaceae are plant pathogens causing leaf and stem lesions (Chen et al., 2017); *Phoma* and *Didymella* are reported as pathogenic on kiwifruit (Kwon et al., 2016; Zou et al., 2020).

To explore the role of dysbiosis in KVDS-affected orchards, bNTI values were calculated. The results showed that fungal community assembly processes were mainly stochastic in nature for both healthy and diseased orchards. For abundant fungal taxa, defined as greater than or equal to 1% relative abundance, Jiao and Lu (2020) found stochastic assembly to be dominant in agricultural fields (maize and rice). Similarly, Xiong et al. (2021) observed a large effect of stochastic processes, matrix, and crop in shaping crop fungal communities of maize, wheat, and barley. These results show a common dynamic of fungal assemblies in agricultural soils.

By comparison, bacterial community assembly was largely driven by stochastic processes in diseased orchards relative to healthy ones. According to Arnault et al. (2023) this finding is consistent with an expected increase in stochastic processes in disease-associated microbiota, leading to higher variability in communities under stress conditions.

For oomycetes, however, deterministic processes had 2.5 times higher impact in diseased communities compared to healthy ones. At the same time, closer inspection of bNTI values (100% bNTI < −2) associated with these processes indicated the presence of the Anti-Anna Karenina Principle (Anti-AKP), in which dysbiosis is regulated mainly by deterministic processes due to modification of the host biology permitting the onset of a novel and potentially pathogenic community (Arnault et al., 2023). Taken together, these findings suggest that different components of the rhizosphere microbiome associated with KVDS undergo an evolution compatible with dysbiotic processes.

The physicochemical properties of soils were integrated into the network analysis to assess their role in changing the plant’s ability to withstand abiotic stress while influencing the plant-associated microbiome (Giovannetti et al., 2023). Similar to grapevine decline, where physicochemical parameters of soils alone could not explain the presence of decline in affected vineyards compared to asymptomatic ones (Darriaut et al., 2021), KVDS-affected and healthy orchards did not cluster differently based on soil physicochemical properties measured in this study. In vineyards, further investigation revealed a dysbiosis phenomenon in microbial communities and dysregulation of ecosystem processes linking soil status to vine fitness (Darriaut et al., 2021).

However, different soil parameters, including assimilated and exchanged minerals, showed indirect (third or higher degree) associations with genera interacting with *Phytopythium* in the rhizosphere. It is also worth noting that health status had a third-degree association with exchanged potassium and assimilated manganese, which are key elements for healthy kiwifruit that influence both fruit set and size (Clark and Smith, 1988) as well as root growth (Buwalda and Hutton, 1988).

## 5 Conclusion

KVDS is a multifactorial syndrome in which multiple biotic components and abiotic stress brought about by climate change act synergically. Bacteria, fungi, and oomycetes were considered together in three different matrices, representing a spatial gradient of the belowground system, sampled in the main production area of Northwestern Italy. Together with the microbial populations associated with the syndrome, the physicochemical parameters of soils were also investigated, revealing their roles in shaping fungal communities. Further analysis of the association networks between soil physiochemical characteristics and rhizosphere communities revealed indirect associations with *Phytopythium* spp. or with the plant health status. These abiotic factors play a role in reshaping the plant microbiota and may promote dysbiosis, especially when the plant is already weakened. The oomycete genera *Phytopythium,* previously reported in several studies as associated with the syndrome (Prencipe et al., 2020; Savian et al., 2021), was present in healthy and diseased fields, albeit in different relative abundances. Association networks of co-occurrence and co-exclusion between taxa were investigated for the first time in this pathosystem, unveiling the correlation of *Phytopythium* spp. with the diseased status of orchards in soils. In the rhizosphere, the oomycete ASVs were negatively associated with saprobes and plant growth-promoting fungal genera such as AM fungi. Network analyses of rhizosphere communities showed bacterial and fungal associations were established with different *P. vexans* ASVs, thus revealing strain-specific characteristics that require further investigation. The dynamic emerging from our analysis of the ecological processes driving rhizosphere community assembly highlights the possibility of a dysbiosis phenomenon driven by Anti-AKP in oomycete communities. Unlike fungal communities and bacterial assemblies in diseased samples that were dominated by stochastic processes. This study highlights the importance of considering multifactorial stressors and their interaction in emerging pathosystems. Environmental variations like flooding and higher temperatures in the soil are likely to favor genera whose lifestyle requires such conditions, and these factors should be included in future analysis. Therefore, a relevant consideration for future studies is the investigation of water management and moisture levels in the soil close to roots and the possible correlation with oomycete community development and soil temperature in the field. Soil enzymatic activity and/or metagenomic studies should also be considered to further investigate the functional role of communities associated with symptomatic orchards.

## Data availability statement

The data presented in the study are deposited in the ENA repository, accession number PRJEB70619.

## Author contributions

MiG: Conceptualization, Investigation, Methodology, Writing – original draft, Writing – review & editing. MaG: Data curation, Formal analysis, Writing – review & editing. LN: Resources, Writing – review & editing. YZ: Writing – review & editing. SD: Writing – review & editing. DS: Conceptualization, Funding acquisition, Methodology, Resources, Supervision, Writing – review & editing.

## Footnotes

^1^ https://www.ncbi.nlm.nih.gov/nucleotide/

^2^ https://servizi.regione.piemonte.it/catalogo/analisi-dei-terreni-altre-analisi-chimiche-agrichim

^3^ https://www.ebi.ac.uk/ena/browser/
