# Supplementary figures and images for: Soil, rhizosphere, and root microbiome in kiwifruit vine decline, an emerging multifactorial disease
Source: Front Microbiol. 2024 Mar 21;15:1330865. doi: 10.3389/fmicb.2024.1330865 (PMC10991698; doi:10.3389/fmicb.2024.1330865)

## Slide 1
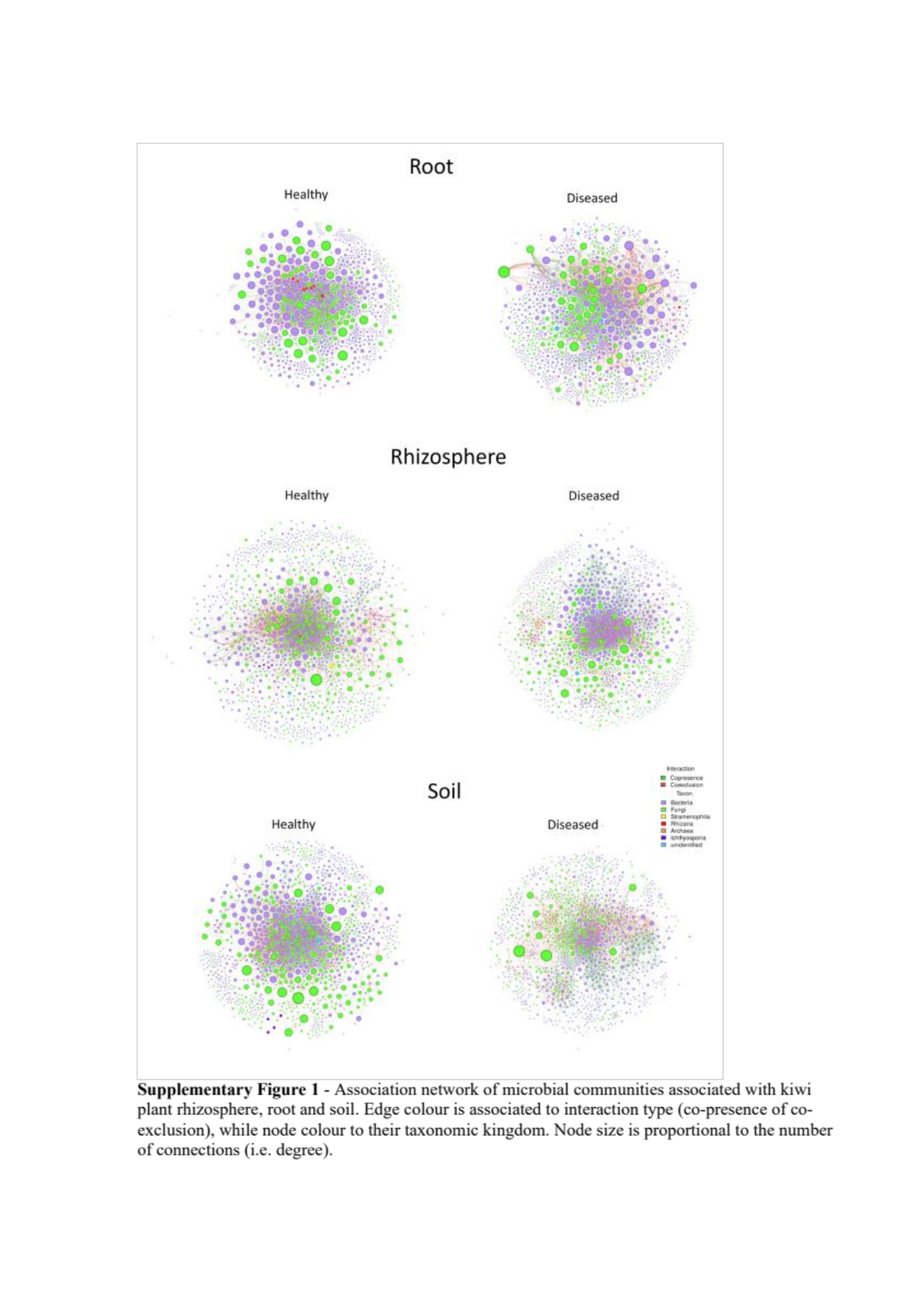

## Slide 2
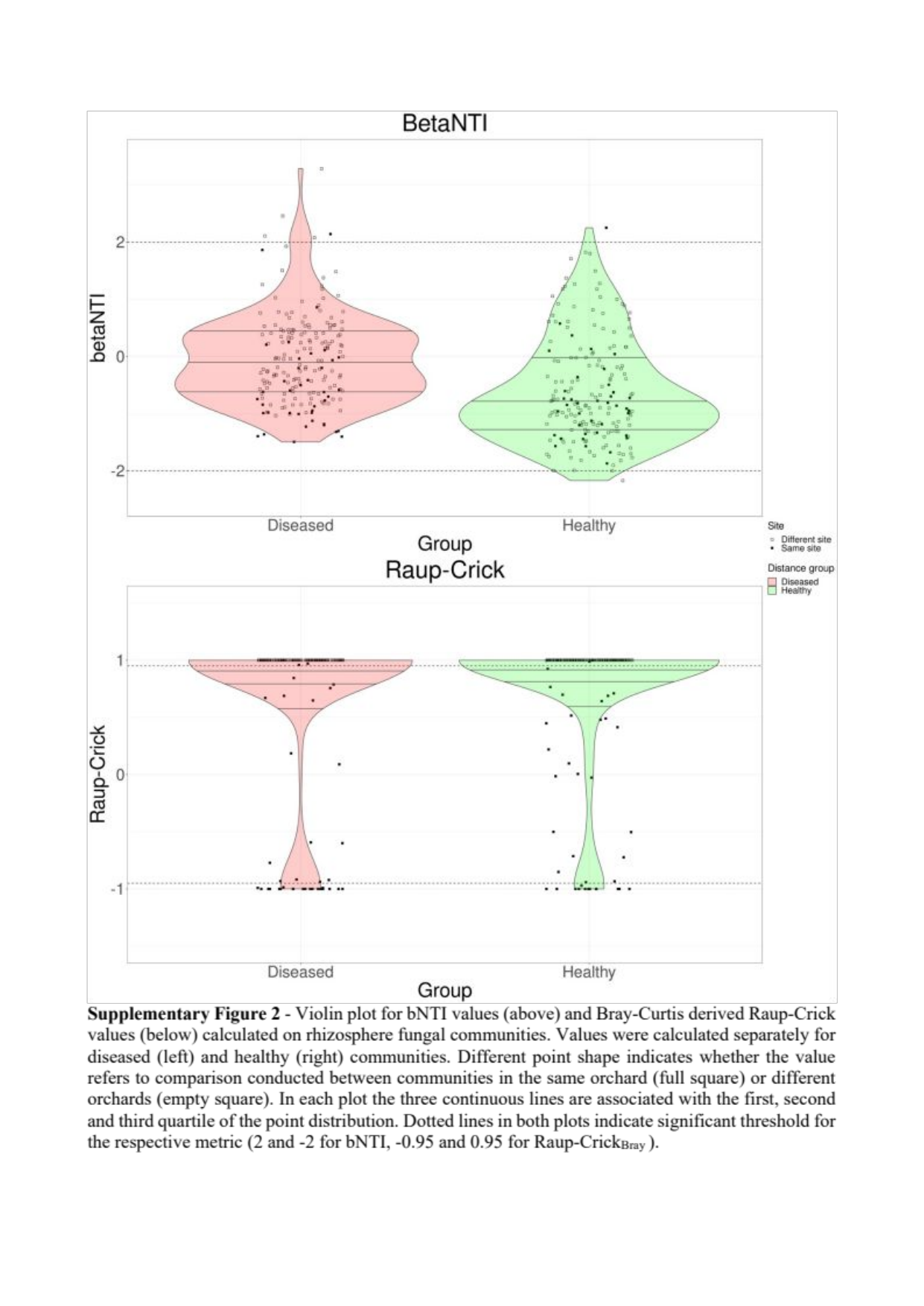

## Slide 3
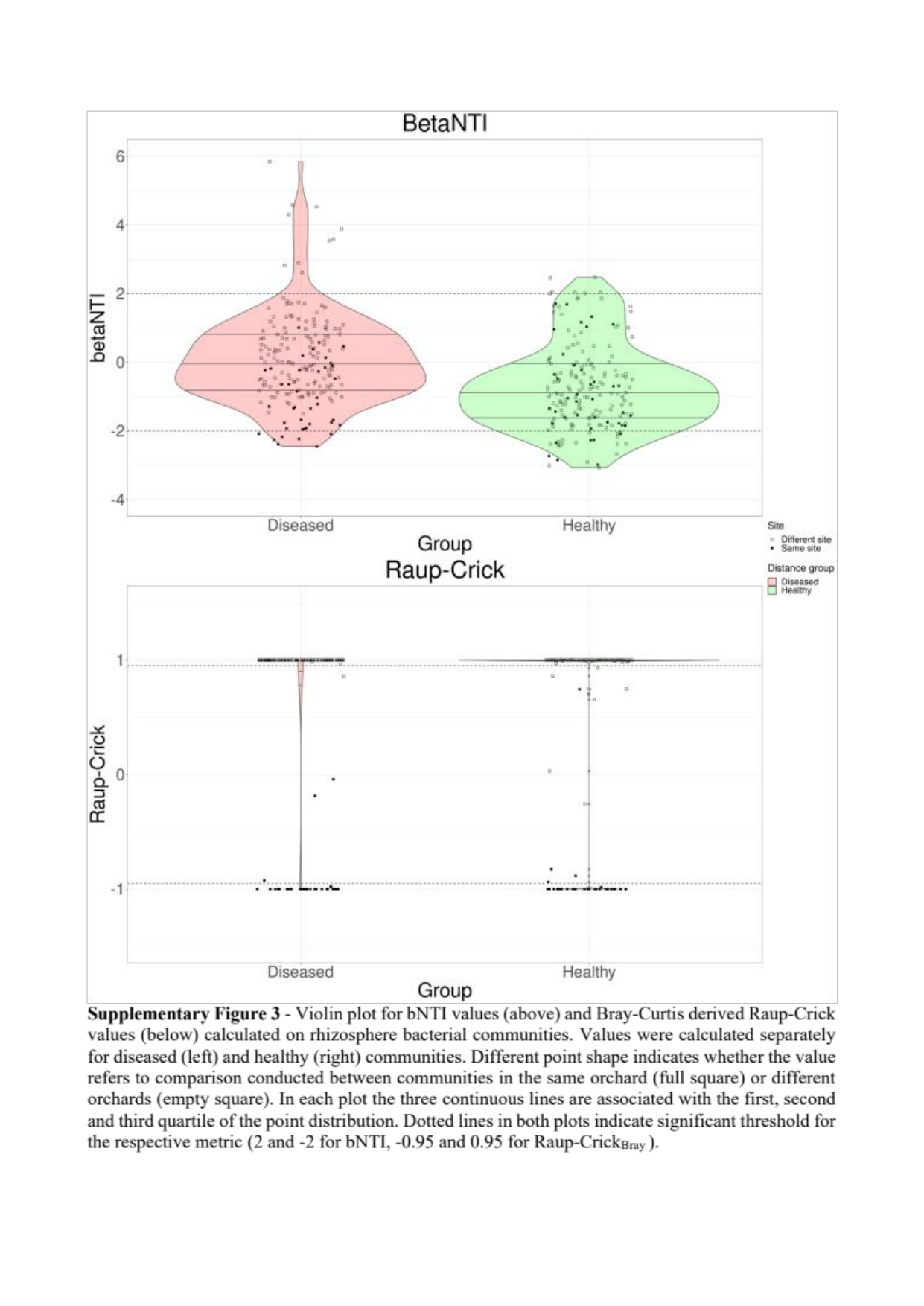

## Slide 4
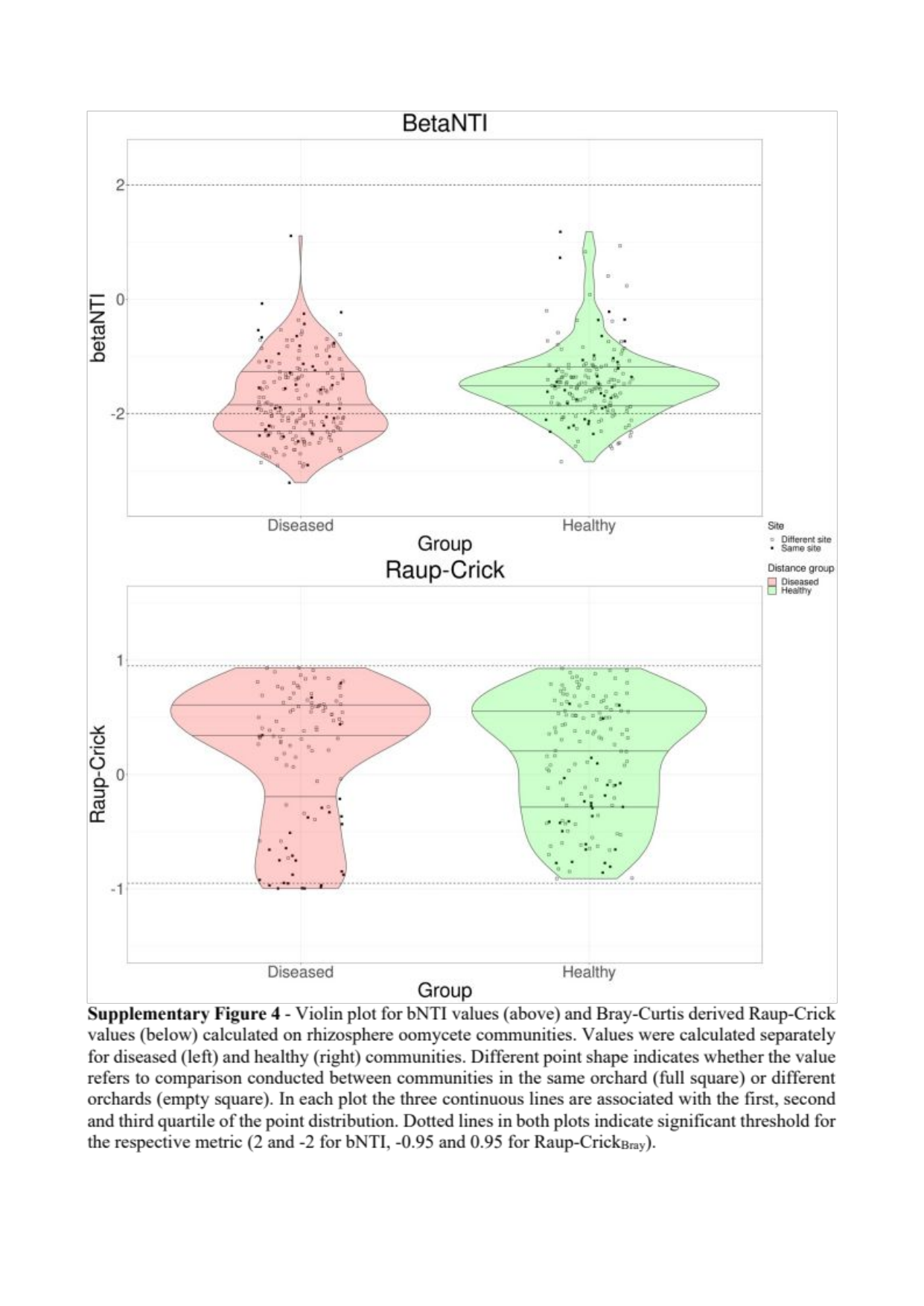

## Slide 5
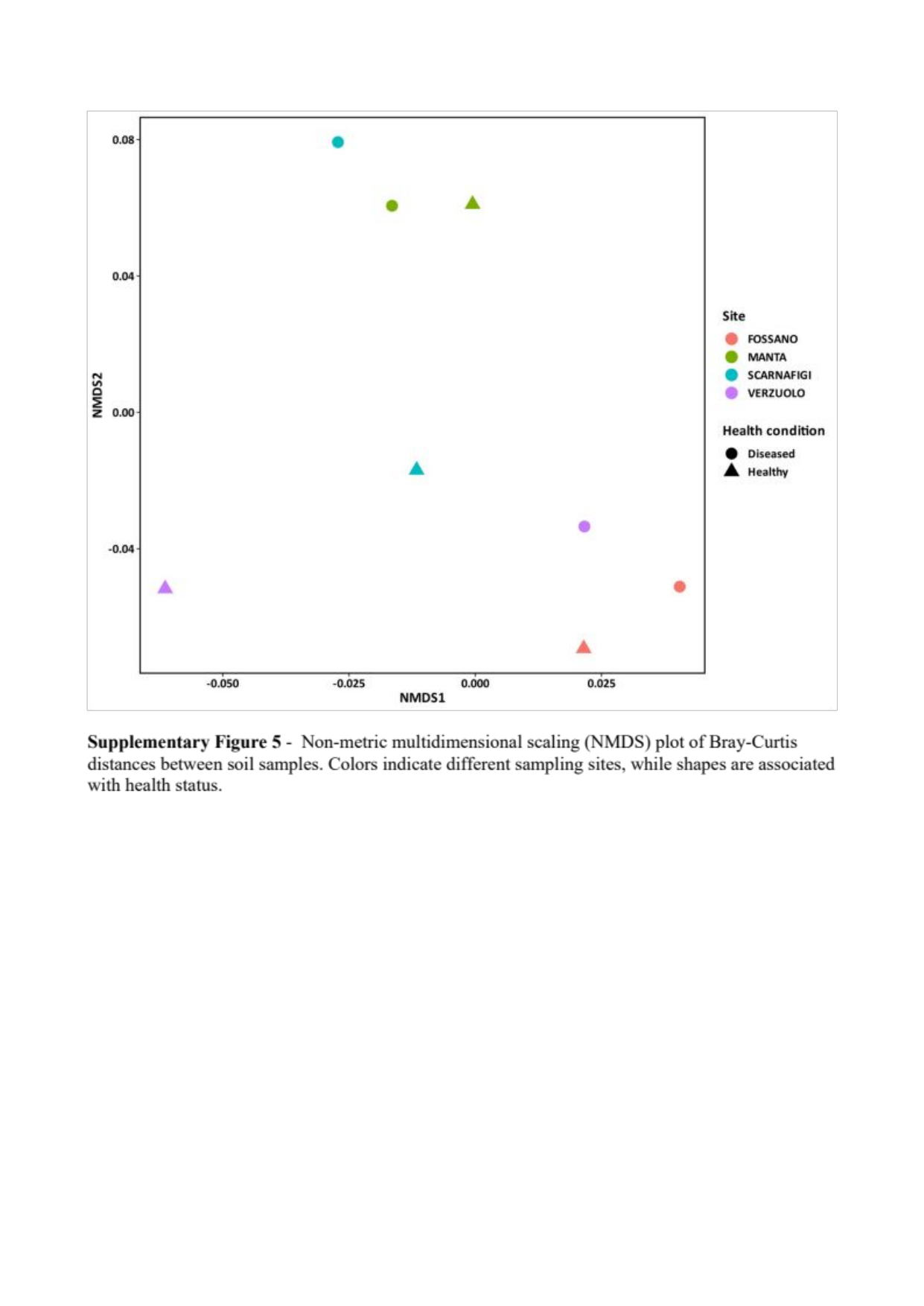

Supplement: Supplementary file 1 [file Presentation_1.PPTX]
